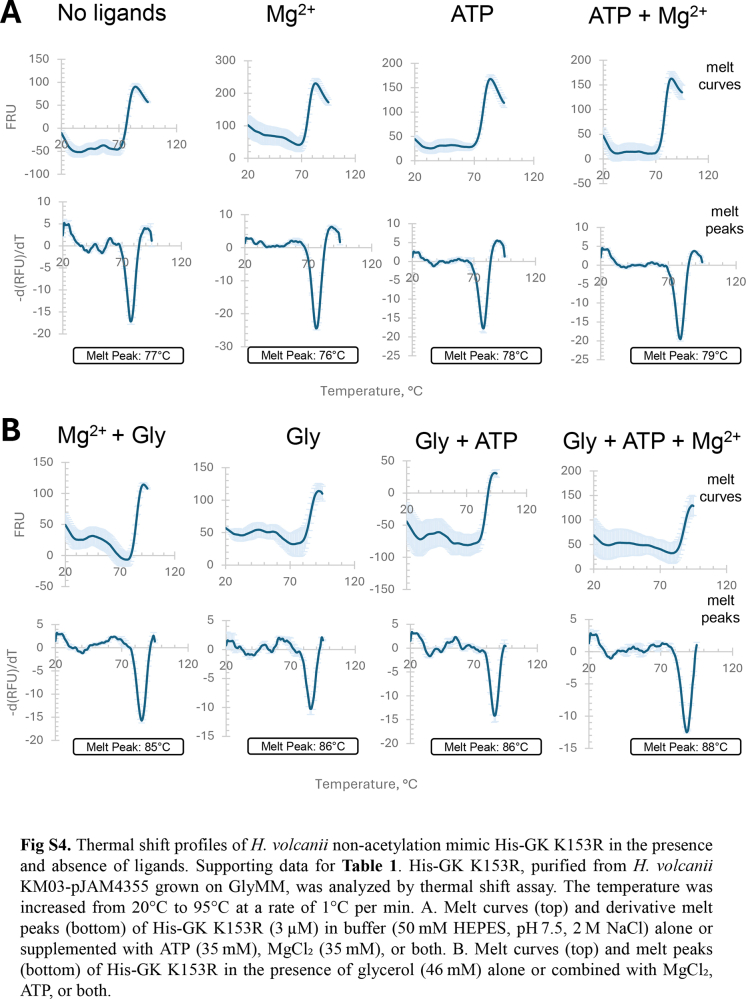# Dynamic acetylation of a conserved lysine impacts glycerol kinase activity and abundance in the haloarchaeon *Haloferax volcanii*

**DOI:** 10.1016/j.jbc.2025.110960

**Published:** 2025-11-20

**Authors:** Karol M. Sanchez, Manasa Addagarla, Heather N. Judd, Xin Wang, Julie A. Maupin-Furlow

**Affiliations:** 1 Department of Microbiology and Cell Science, Institute of Food and Agricultural Sciences, University of Florida, Gainesville, Florida, USA; 2 Genetics Institute, University of Florida, Gainesville, Florida, USA

**Keywords:** lysine acetylation, carbon source adaptation, metabolic regulation, post-translational modification, glycerol metabolism, glycerol kinase, protein stability, hypersaline, archaea

## Abstract

Lysine acetylation is a key regulator of metabolism, but its role in archaeal carbon metabolism remains unclear. In halophilic archaea such as *Haloferax volcanii*, glycerol kinase (GK, *glpK*) catalyzes the phosphorylation of glycerol to glycerol-3-phosphate, the first committed step in glycerol catabolism. Unlike bacterial GKs, which are typically repressed during glucose metabolism, *H*. *volcanii* prefers glycerol as a carbon and energy source rather than glucose, suggesting that GK across species is regulated by distinct mechanisms. Here, we show that lysine acetylation enhances *H*. *volcanii* GK (*Hv*GK) activity, allosteric behavior, stability, and cellular abundance during growth on glycerol. Lysine residue 153 (K153), located within a conserved flexible loop, was identified as the primary acetylation site and reached up to 78% acetylation occupancy in glycerol-grown cells, as determined by AQUA-MS. Shifting from glucose to glycerol increased both *Hv*GK activity and K153 acetylation. Functional assays revealed that *Hv*GK and the acetylation mimic variant K153Q supported growth on glycerol, while the nonacetylatable variant K153R did not. The K153R substitution also reduced protein stability, as shown by thermal shift assays, and altered cooperative substrate binding behavior. The GNAT-family acetyltransferase Pat2 was found responsible for acetylating *Hv*GK at K153. In *Δpat2* mutants, *Hv*GK levels were significantly reduced but rescued by the K153Q variant, indicating acetylation protects *Hv*GK from degradation. Together, these findings reveal that lysine acetylation dynamically coordinates *Hv*GK structure and function in response to carbon source availability, positioning acetylation as a key posttranslational mechanism of metabolic control in *H*. *volcanii*.

Glycerol metabolism plays a critical role in the survival and environmental adaptation of haloarchaea in hypersaline environments (1). In *Haloferax volcanii*, glycerol is the preferred carbon source over glucose, with glycerol kinase (GK) serving as the key enzyme for glycerol utilization. *H*. *volcanii* GK (*Hv*GK) catalyzes the phosphorylation of glycerol to glycerol-3-phosphate using MgATP as the phosphoryl donor, linking glycerol assimilation to central carbon metabolism. (2). Our recent work characterized the biochemical properties of the *Hv*GK enzyme, demonstrating its robust catalytic activity under extreme conditions, including exposure to organic solvents, elevated temperatures, and high salinity (3). However, the regulatory mechanisms governing *Hv*GK activity in *H*. *volcanii* remained unknown.

Distinct regulatory mechanisms appear to govern bacterial and archaeal GKs. In bacteria, such as *Escherichia coli*, GK activity is allosterically inhibited by fructose-1,6-bisphosphate and the EIIA component of the phosphotransferase system, both of which accumulate during glycolytic metabolism when intracellular acetate levels are low (4, 5, 6). In contrast, archaeal GKs, including *Hv*GK, lack the corresponding regulatory domains found in their bacterial counterparts, suggesting that archaea have evolved alternative strategies to modulate GK activity in response to carbon source availability.

Upon exposure to glycerol, glucose catabolism in *H*. *volcanii* is repressed at both transcriptional and protein levels, indicating a carbon catabolite repression mechanism adapted to favor glycerol metabolism (7). This prioritization is enforced at the transcriptional level by GlpR, a DeoR-family repressor, which downregulates the expression of genes involved in glucose and fructose catabolism in the presence of glycerol (7). In contrast, the transcriptional regulator GfcR acts as an activator of sugar catabolism when glycerol is limited, promoting the expression of genes encoding enzymes involved in glucose and fructose degradation, including gluconate dehydratase, glyceraldehyde-3-phosphate dehydrogenase, and pyruvate kinase (8). Despite these insights, the regulatory mechanism governing the activation of glycerol metabolism is not yet fully understood.

Beyond transcriptional regulation, recent omics approaches have expanded the understanding of post-translational modifications (PTMs) in *H*. *volcanii*, including lysine acetylation. A high-throughput mass spectrometry (MS) study of glycerol-grown *H*. *volcanii* has identified *Hv*GK to be acetylated at residue K153, suggesting a potential role for lysine acetylation in the regulation of carbon metabolism (9). Acetylation at the analogous lysine residue is also reported in the GK of *Haloferax mediterranei*, implying conservation of this PTM across halophilic archaea (10).

Acetylation can influence protein conformation, stability, solubility, interactions, and enzymatic activity by altering surface charge and hydrophobicity (11). Lysine acetylation can function as a reversible regulatory mechanism, capable of either activating or repressing enzyme activity depending on the context (12). Lysine acetylation is best known for its role in modifying histones in eukaryotes (13); however, its functions are far more diverse, with the full range of targets only recently being uncovered.

Lysine acetylation is an evolutionarily conserved PTM that is dynamically modulated in response to nutrient availability, acting as a metabolic rheostat (14, 15) that is associated with stress responses and survival (16, 17). Changes in the abundance of lysine acetylated proteins and their associated PTM sites during nutrient transitions are well documented in bacteria (18, 19, 20) and eukaryotes (21, 22). Lysine acetylation is found to modulate the activity of key enzymes that direct carbon flux between glycolysis and gluconeogenesis, as well as the partitioning of metabolites between the trichloroacetic acid cycle and the glyoxylate shunt (23). Lysine acetylation also influences the levels of central metabolites including ATP, NAD^+^, and acetyl-CoA (24), highlighting that metabolic shifts are actively regulated rather than passively altered (16, 25).

Although lysine acetylation is increasingly recognized as a key regulator of metabolism, its role in central carbon metabolism remains poorly understood in archaea. Likewise, the mechanisms controlling archaeal GKs, which differ from those in bacteria, have not been defined. In this study, we show that lysine acetylation enhances the activity, allosteric regulation, thermal stability, and abundance of *Hv*GK in a carbon source-dependent manner. Pat2-mediated acetylation of a conserved lysine residue (K153) within a flexible loop of *Hv*GK during glycerol metabolism exerts these multiple positive effects and promotes growth on glycerol. The strong conservation of K153 and its surrounding loop region suggests an evolutionarily conserved regulatory mechanism. Collectively, these findings uncover a previously unrecognized post-translational mechanism that controls archaeal metabolism, with broad implications for both biotechnology and evolutionary biology.

## Results

### Lysine acetylation of H*v*GK

**Figure 1:**
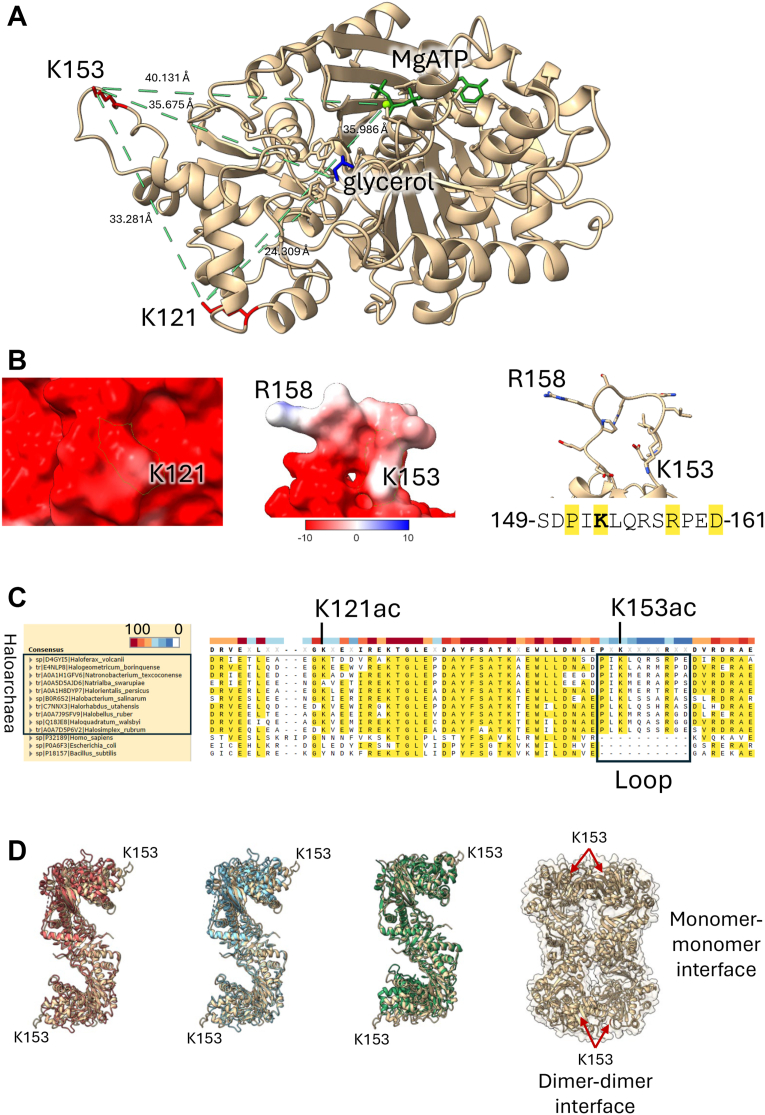
**Structural and evolutionary features of lysine acetylation sites in *Hv*GK**. *A*, predicted 3D-structure of *Hv*GK with ligand binding and Kac sites highlighted. AlphaFold3-predicted 3D-structure of the monomeric unit of the *Hv*GK (UniProt D4GYI5, tan ribbon) bound to MgATP (*green*) at confidence metric scores of ipTM = 0.94 pTM = 0.92. Lysine residues K121 and K153, identified as acetylated by LC-MS/MS in glycerol-grown cells, are indicated (*red*). Glycerol (*blue*)-binding pocket predicted by comparison to *Thermococcus kodakarensis* GK structure (PDB: 6K79) at RMSD between 449 pruned atom pairs of 0.855 Å; (across all 488 pairs: 1.541 Å). Distances between K121, K153, and the bound glycerol and Mg ions are indicated. *B*, close-up view of K121 and K153 acetylation sites. *Left*: Predicted location of K121 within a surface-exposed acidic patch; electrostatic surface shown with K121 highlighted in *green*. *Middle*: Predicted position of K153 in a surface-exposed flexible loop of neutral-to-positive charge; electrostatic surface with K153 highlighted in *green*. *Right*: Structural context of K153 within the flexible loop; amino acids and side chains shown in *stick* representation and colored by heteroatom. *C*, multiple sequence alignment showing conservation of the K153 residue and the loop insertion among haloarchaea (class *Halobacteria*). GK homologs from haloarchaea were compared to those from *Homo sapiens* and bacteria (*Escherichia coli* and *Bacillus subtilis*). Sequences used for alignment are provided in Dataset S1, with representative haloarchaeal GK sequences displayed. *Yellow* highlights indicate residues with a consensus >50%, and the consensus scale ranges from 0 (*white*) to 100 (*red*). *D*, predicted context of K153 in *Hv*GK dimer and tetramer assemblies. AlphaFold model of the *Hv*GK dimer (*beige*; ipTM = 0.77; pTM = 0.82) aligns closely with experimental GK dimers: – *T*. *kodakarensis* GK (*turquoise*; PDB 6K78): sequence alignment score = 1617.4; RMSD = 0.806 Å (444 pruned atom pairs; 1.482 Å across 482 pairs). – *C*. *thermophilum* GK (*red*; PDB 6ZQ8): sequence alignment score = 1399.8; RMSD = 0.979 Å (430 pruned; 1.953 Å across 491 pairs). – *T*. *brucei gambiense* GK (*green*; PDB 6J9X): sequence alignment score = 1422.8; RMSD = 0.834 Å (431 pruned; 2.191 Å across 495 pairs). Across all alignments, *Hv*GK K153 lies > 24 Å from catalytic residues and outside the dimer interface. An exploratory tetramer model (far right, low confidence; ipTM = 0.34, pTM = 0.48) places K153 near an interdimer contact rather than within the active site, consistent with biochemical evidence that tetramer formation occurs under high-acetylation, glycerol-dependent conditions. DTT, dithiothreitol; GK, glycerol kinase; *Hv*GK, *H*. *volcanii* GK; *Hv*GK_Ec,_*E*. *coli*-expressed recombinant His-*Hv*GK; IB, immunoblotting; MS, mass spectrometry; PTM, post-translational modification; SEC, size-exclusion chromatography; wt, wildtype.

High-throughput proteomic analysis previously revealed that *Hv*GK is acetylated at K153 in glycerol-grown cells (9). To expand on this finding, we purified the enzyme from glycerol-grown *H*. *volcanii* cells and mapped the acetylation sites by LC-MS/MS. Two sites of acetylation (K121 and K153) were observed in the purified enzyme. To gain insight into how acetylation of these lysine residues may influence enzyme structure and activity, we used 3D-modeling to predict the orientation of K153 and K121 with respect to the MgATP and glycerol binding sites (Fig. 1*A*). Both lysine residues were found on the protein surface at a substantial distance from the substrate binding pocket. Considering that haloarchaea produce proteins with highly acidic surfaces that help maintain proper folding and function in the high-salt cytosol (26), we performed Coulombic surface electrostatic modeling (Fig. 1*B*). Despite its basic nature, K121 was found to reside in a strongly acidic surface patch, contributing little to the net charge of the region (Fig. 1*B*, *left*). In contrast, K153 was predicted to be in a neutral-to-positively charged flexible loop (Fig. 1*B*, *middle-right*), suggesting that the acetylation of this surface exposed lysine residue may modulate protein interactions and/or conformational dynamics, potentially at intersubunit interfaces rather than within the same monomer. Multiple sequence alignment of *Hv*GK homologs showed that K153-containing loop is highly conserved among haloarchaea, present in 36 of 41 Halobacteria homologs (≈88%) and absent in only three strains likely misclassified within the group. In contrast, K121 is less conserved, retained in roughly 73% of sequences. Moreover, K121, K153, and the loop were not found to be conserved in bacterial or eukaryal homologs (Fig. 1*C*). These findings suggest acetylation of lysine 153 represents a haloarchaeal-specific adaptation that may fine-tune intersubunit interactions or conformational flexibility to optimize *Hv*GK function under hypersaline conditions. To further contextualize this residue, we modeled *Hv*GK as a dimer and compared it to experimentally determined GK dimers, revealing that K153 lies > 24 Å from the active site and outside the dimer interface but near the interdimer contact observed in low-confidence tetramer models (Fig. 1*D*).

### K153 is the major lysine acetylation site of H*v*GK

**Figure 2:**
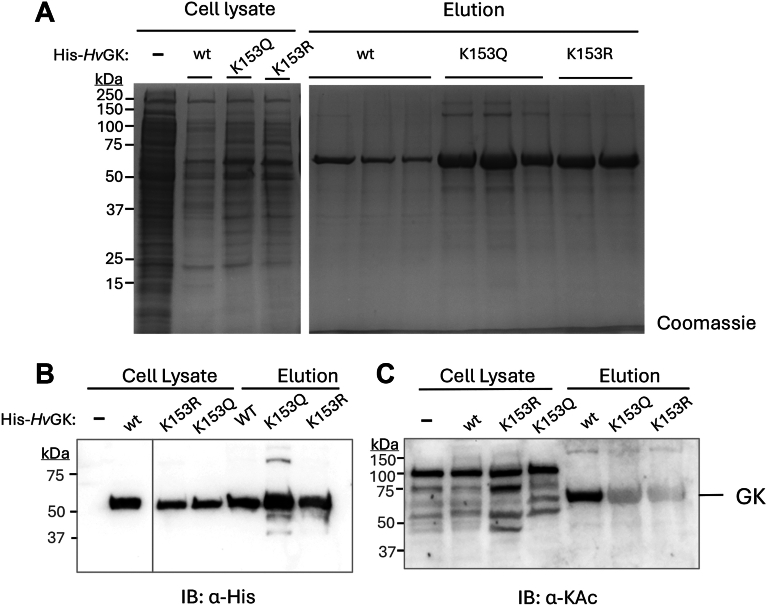
**Detection of K153 as the primary acetylation site in *Hv*GK**. *A*, purification of *Hv*GK enzymes. *H*. *volcanii* H1207 strains carrying plasmids expressing His-*Hv*GK wt, K153Q and K153R, or an empty vector control (−) were grown to stationary phase in ATCC 974 medium. Cells were lysed (cell lysate), and proteins were purified by Ni-NTA resin (elution). Protein samples were separated by 12% SDS-PAGE and stained with CB. *B*, detection of His-tagged *Hv*GK proteins. Samples analyzed by immunoblotting using anti-His antibodies (IB: α-His) to assess the abundance of His-tagged *Hv*GK enzymes in the elution fractions. *C*, analysis of lysine acetylated proteins. Protein samples were analyzed by immunoblotting using anti-acetyllysine antibodies (IB: α-KAc) to compare the lysine acetylated state of proteins in the cell lysate and Ni-NTA elution fractions. All experiments were performed in biological triplicates. Representative immunoblots are shown. CB, Coomassie blue; GK, glycerol kinase; *Hv*GK, *H*. *volcanii* GK.

To assess the impact of K153 on the overall levels of lysine-acetylated *Hv*GK, amino acid substitutions K153R and K153Q were generated. The K153Q variant represents a charge-neutral substitution commonly used as an acetylation mimic, approximating the electrostatic and hydrogen-bonding properties of acetylated lysine, while K153R retains the positive charge and serves as a nonacetylatable control. N-terminally His-tagged wildtype (wt) and variant *Hv*GKs were expressed and purified by Ni-NTA chromatography to apparent homogeneity (>90% purity, assessed by SDS-PAGE). Cells were grown in ATCC 974 rich medium (yeast extract and tryptone) to establish baseline acetylation levels independent of defined carbon source conditions. SDS-PAGE analysis showed that wt and variant *Hv*GKs exhibited similar expression levels and protein yields (Fig. 2*A* and *B*). Coomassie blue (CB) staining and immunoblotting (IB) for the His-tag and acetyl-lysine epitopes revealed that wt *Hv*GK displayed a strong lysine acetylation signal, which was markedly reduced in both the K153R and K153Q variants (Fig. 2*C*). These data indicate that K153 is the predominant lysine acetylation site of *Hv*GK under nutrient-rich growth conditions.

### Pat2 acetylates K153 of H*v*GK

**Figure 3:**
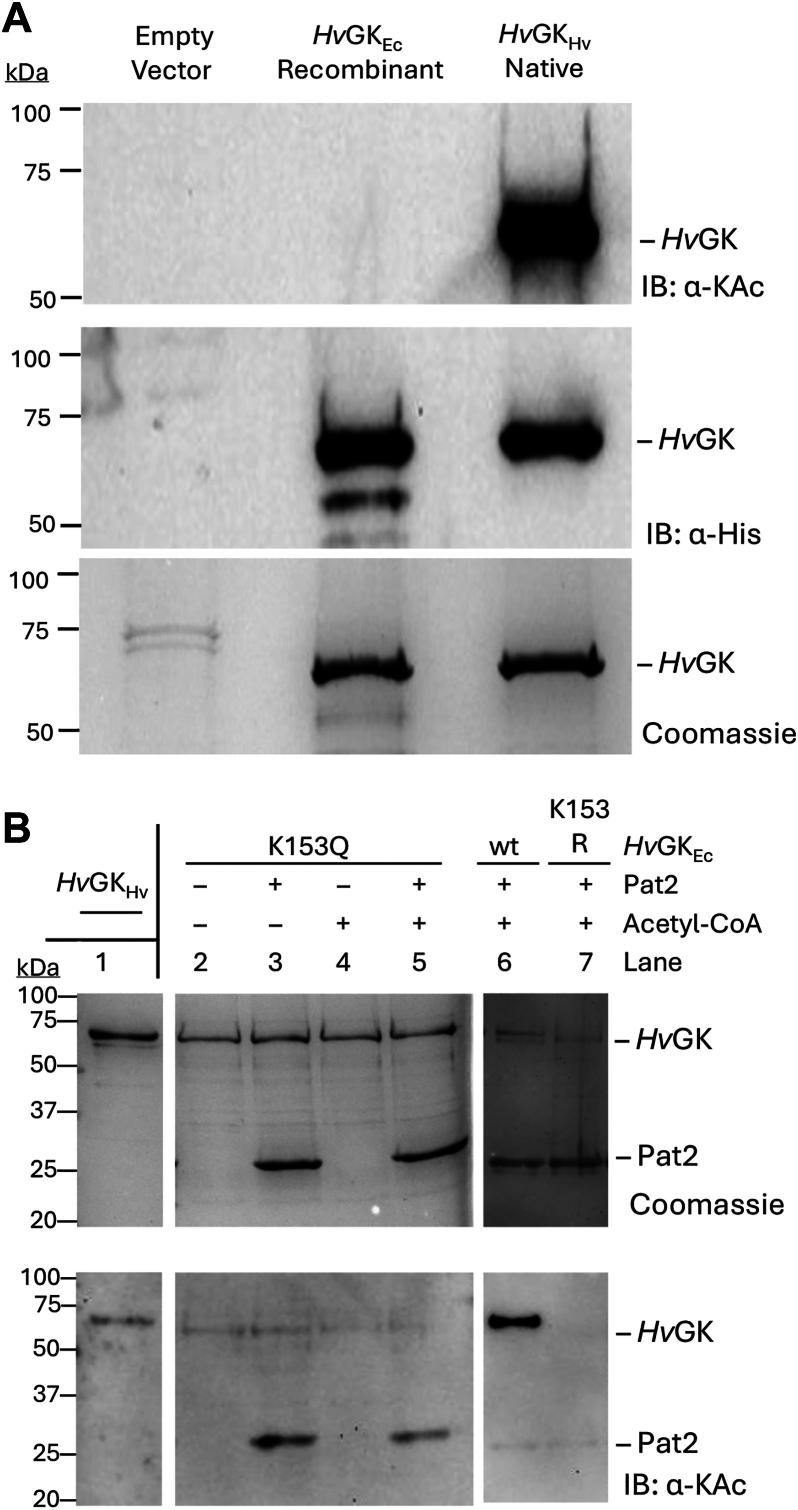
**Pat2 selectively acetylates the K153 site of *Hv*GK**. *A*, absence of the lysine acetylation of *Hv*GK when expressed and purified from recombinant *E*. *coli*. Proteins were purified by Ni-NTA chromatography from *H*. *volcanii* H1207 strains expressing His-*Hv*GK from a plasmid (*Hv*GK_Hv_) or the empty vector control compared to recombinant *E*. *coli* expressing the His-*Hv*GK enzyme (*Hv*GK_Ec_). Total protein abundance in sample was detected by CB staining. His-*Hv*GK enzyme was detected by IB: α-His. Lysine acetylation levels were assessed by IB: α-KAc. *B*, *in vitro* acetylation of *Hv*GK_Ec_ wt compared to the K153 variant proteins. Reactions included 2 μM of *Hv*GK_Ec_ (wt, K153Q or K153R) incubated with 6 μM Pat2 acetyltransferase and 0.2 mM acetyl-CoA, as indicated. Acetylation was detected by IB: α-KAc. CB staining confirmed equal protein loading. *Hv*GK_Hv_ was directly applied to the SDS-PAGE gels to compare its migration to the *Hv*GK_Ec_ enzymes. All experiments were performed in biological triplicates. Representative immunoblots are shown. CB, Coomassie blue; GK, glycerol kinase; *Hv*GK, *H*. *volcanii* GK; IB: α-His, immunoblotting using anti-His antibodies; IB: α-KAc, immunoblotting using anti-acetyllysine antibodies.

To independently determine the specificity of K153 acetylation, *in vitro* acetylation assays were performed using the acetyltransferase Pat2, previously reported to acetylate *Hv*GK (27). Recombinant nonacetylated *E*. *coli*-expressed His-*Hv*GK (*Hv*GK_Ec_) proteins (wt, K153Q, and K153R) were purified for use in these assays. After purification, the identity and nonacetylated status of the recombinant *Hv*GK_Ec_ wt protein was verified by LC-MS/MS analysis and IB (Fig. 3*A*). For the *in vitro* assay, equal concentrations of the purified *Hv*GK_Ec_ wt and variants (Fig. 3*B*) were incubated with Pat2 in the presence of acetyl-CoA as the acetyl group donor. Lysine acetylation was then assessed by IB (IB: α-KAc, Fig. 3*B, lower panel*). In agreement with the *in vivo* results, only *Hv*GK_Ec_ wt was acetylated by Pat2, while the K153Q and K153R variants remained unmodified (Fig. 3*B*). These findings identify K153 as the primary site of lysine acetylation on *Hv*GK and confirm that acetylation at this site is mediated by Pat2.

### Lysine acetylation of H*v*GK modulated by carbon source availability

**Figure 4:**
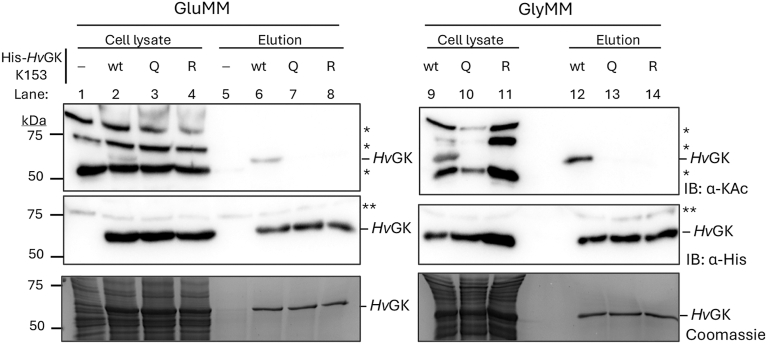
**K153-acetylated *Hv*GK, the major acetylated form of this enzyme, appears elevated in glycerol- *versus* glucose-grown cells**. His-*Hv*GK and variant proteins were purified by Ni-NTA affinity chromatography from *H*. *volcanii* KM03 (Δ*glpK* Δ*larC*) strains expressing His-*Hv*GK wt, K153Q, or K153R that were grown in GluMM or GlyMM, as indicated. GluMM-grown KM03 carrying the empty vector (pJAM202c, indicated by ─) was included as a control; this strain did not grow in GlyMM due to the essential role of *Hv*GK under these conditions. IB: α-His and CB staining were used to determine the abundance of the His-tagged proteins and confirm equal protein loading, respectively. IB: α-KAc was used to examined lysine acetylation. Clarified lysates (Ni-NTA input) were diluted 1:10, and all elution fractions were normalized to 0.5 μg protein. Theoretical molecular weight of *Hv*GK is 56.7 kDa. *Asterisks*: (∗) lysine acetylated proteins detected in cell lysate in addition to *Hv*GK; (∗∗) nonspecific signal detected with anti-His antibody in empty vector control. All experiments were performed in biological triplicates. Representative immunoblots are shown. ATCC, tryptone/yeast extract; CB, Coomassie blue; FruMM, fructose minimal medium; GK, glycerol kinase; GluMM, glucose minimal medium; *Hv*GK, *H*. *volcanii* GK; IB: α-His, immunoblotting using anti-His antibodies; IB: α-KAc, immunoblotting using anti-acetyllysine antibodies.

**Figure S1:**
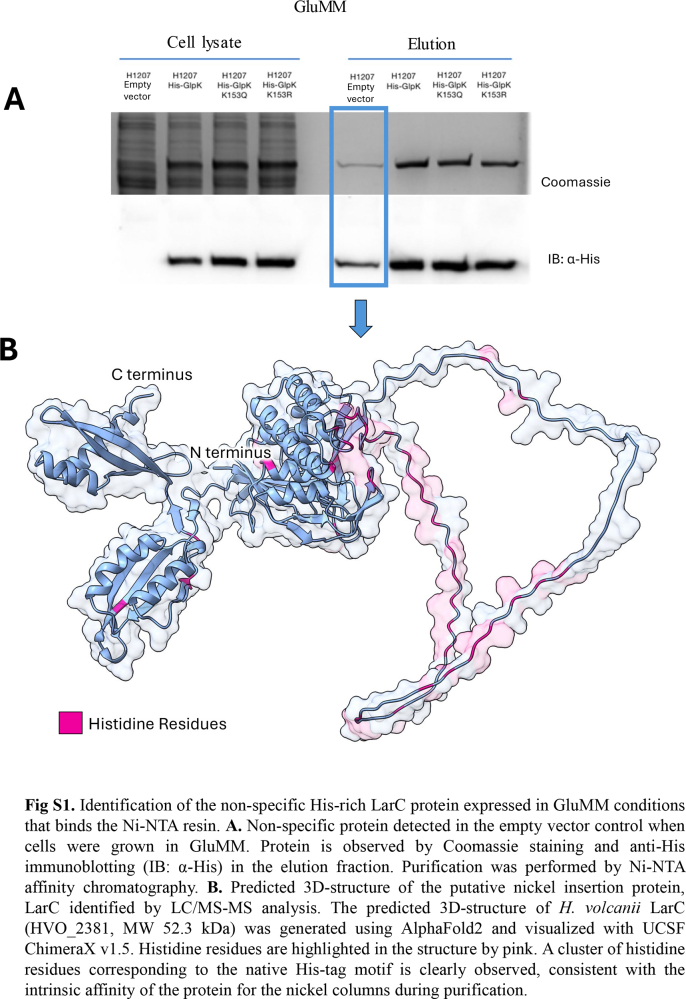


To determine whether *Hv*GK acetylation responds to carbon source changes, we compared the acetylation state of His-tagged *Hv*GK expressed in *H*. *volcanii* grown with either glucose or glycerol. To avoid interference from a native histidine-rich protein (LarC) identified by MS in glucose-grown cells, an engineered *H*. *volcanii* strain lacking both *glpK* and *larC* (KM03) was used for expression (Fig. S1). His-tagged *Hv*GK proteins (wt, K153Q and K153R) were purified from this strain, and their acetylation status was analyzed by IB (Fig. 4). His-*Hv*GK wt showed markedly higher acetylation when cells were grown on glycerol compared to glucose (*lane* *12* *versus lane* *6*) (Fig. 4). The lysine acetylation signal was specific to K153, as no detectable acetylation was observed for the K153Q (*lanes 7 and 13*) or K153R (*lanes 8 and 14*) variants (Fig. 4). These results demonstrate that acetylation at lysine 153 increases during growth on glycerol, indicating that this modification is responsive to carbon source availability and that the KM03 strain provides a reliable background for assessing this effect.

### Carbon source shift reveals dynamic acetylation and its functional impact

**Figure 5:**
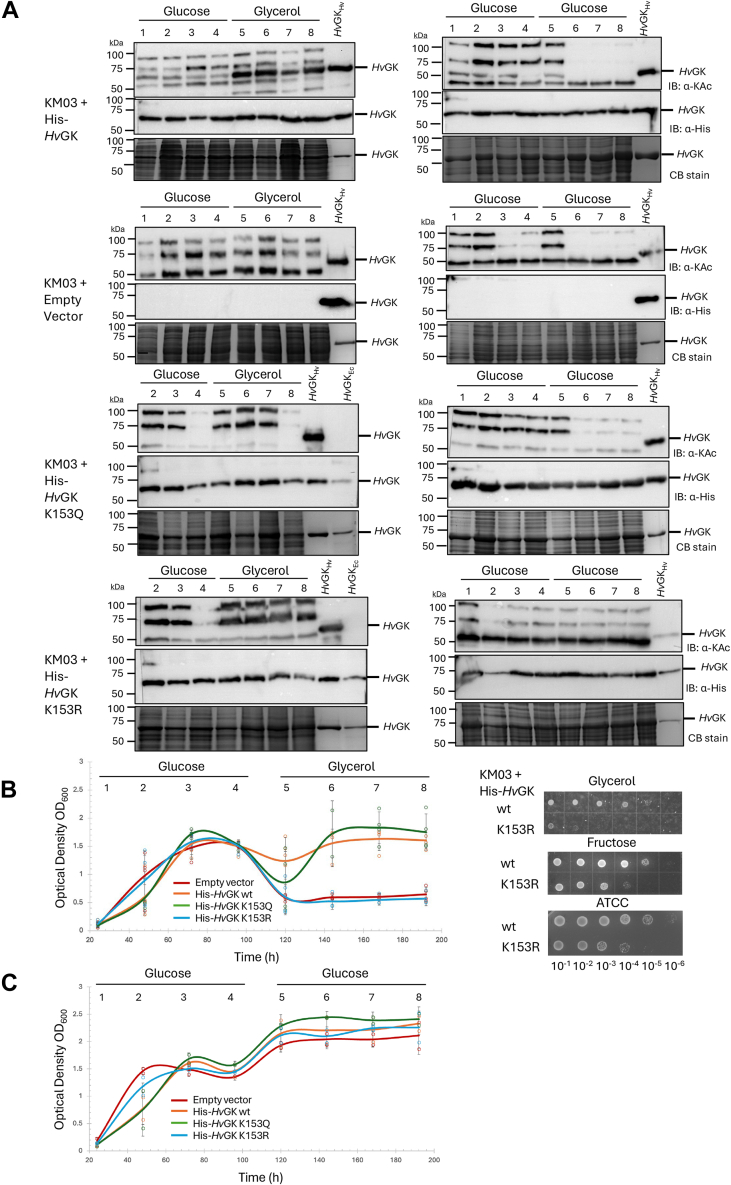
**Increased abundance of K153-acetylated *Hv*GK after glucose-to-glycerol shift and growth defect of the nonacetylatable K153R mimic highlight a positive role for lysine acetylation in glycerol metabolism**. *A*, comparison of lysine acetylome profiles and the abundance of K153-acetylated *Hv*GK following a glucose-to-glycerol shift (*left*) *versus* a glucose-to-fresh glucose transfer (*right*). *H*. *volcanii* KM03 strains constitutively expressing His-tagged *Hv*GK wildtype (wt), K153Q, K153R, or an empty vector (indicated far *left*) were grown in glucose minimal medium (GluMM) for 4 days, then either shifted to glycerol minimal medium (GlyMM, *left*) or transferred to fresh GluMM (right) for an additional 4 days. Lane numbers represent time points as days: 1 (24 h), 2 (48 h), 3 (72 h), 4 (96 h), 5 (120 h), 6 (144 h), 7 (168 h), and 8 (192 h). Whole-cell lysates were collected daily, separated by SDS-PAGE, and analyzed by Coomassie blue (CB) staining and immunoblotting (IB) using anti-acetyllysine (α-KAc) and anti-His (α-His) antibodies, as indicated on the *right*. Purified His-*Hv*GK from *H*. *volcanii* (*Hv*GK_Hv_) and recombinant *E*. *coli* (*Hv*GK_Ec_) served as positive controls for α-His, and as positive and negative controls, respectively, for α-KAc detection. *B*, growth and viability of *H*. *volcanii* strains following the glucose-to-glycerol shift. *Left*: Growth (see *panel A* for details) was monitored by optical density at 600 nm (OD_600_). *Right*: Viability was assessed by spot plating 5 μl serial dilutions on glycerol (GlyMM), fructose (FruMM), and rich medium (ATCC 974) after the carbon source shift (192 h samples). *C*, growth of *H*. *volcanii* strains following glucose-to-fresh glucose transfer. See *panels A* and *B* for experimental details. All experiments were performed in biological triplicates. Representative immunoblots are shown. ATCC, tryptone/yeast extract; FruMM, fructose minimal medium; GK, glycerol kinase; *Hv*GK, *H*. *volcanii* GK.

To examine how *Hv*GK acetylation responds to metabolic transitions, *H*. *volcanii* KM03 strains expressing His-*Hv*GK wt, the acetylation-mimic K153Q, the nonacetylatable K153R, or carrying an empty vector were analyzed during shifts between glucose and glycerol media. Analysis of whole-cell lysates showed a general decrease in overall lysine acetylation levels across all strains during prolonged growth in after shift from glucose to glucose, whereas lysine acetylation levels remained relatively stable and increased on the number of bands detected during growth after shift from glucose to glycerol (Fig. 5*A*, *lanes 6–8*, right *versus* left panels). Focusing specifically on K153-acetylated *Hv*GK, its abundance was found to increase following the shift from glucose to glycerol (Fig. 5*A*, KM03 + His-*Hv*GK, left panel, *lanes 5–8*). In contrast, when cells were transferred from stationary-phase glucose cultures into fresh glucose medium, K153-acetylated *Hv*GK levels declined sharply to undetectable levels (Fig. 5*A*, KM03 + His-*Hv*GK, right panel, *lanes 6–8*). Further analysis of growth revealed that following the shift from glucose to glycerol, cells expressing His-*Hv*GK wt or the K153Q variant continued to grow, whereas the K153R-expressing and empty vector strains exhibited growth arrest (Fig. 5*B*, *left*). Spot plating showed that the K153R strain had reduced viability on glycerol compared to the *Hv*GK wt strain and to its growth on other carbon sources (Fig. 5*B*, *right*). In contrast, when transferred from glucose into fresh glucose medium, all four strains exhibited comparable growth with no significant differences observed at the end of the timecourse (Fig. 5*C*). Together, these results demonstrate that *Hv*GK acetylation is dynamically regulated by carbon source availability and that acetylation of lysine 153 enhances growth and metabolic adaptation under glycerol conditions.

### H*v*GK acetylation levels correlate with its catalytic activity across carbon sources

**Figure 6:**
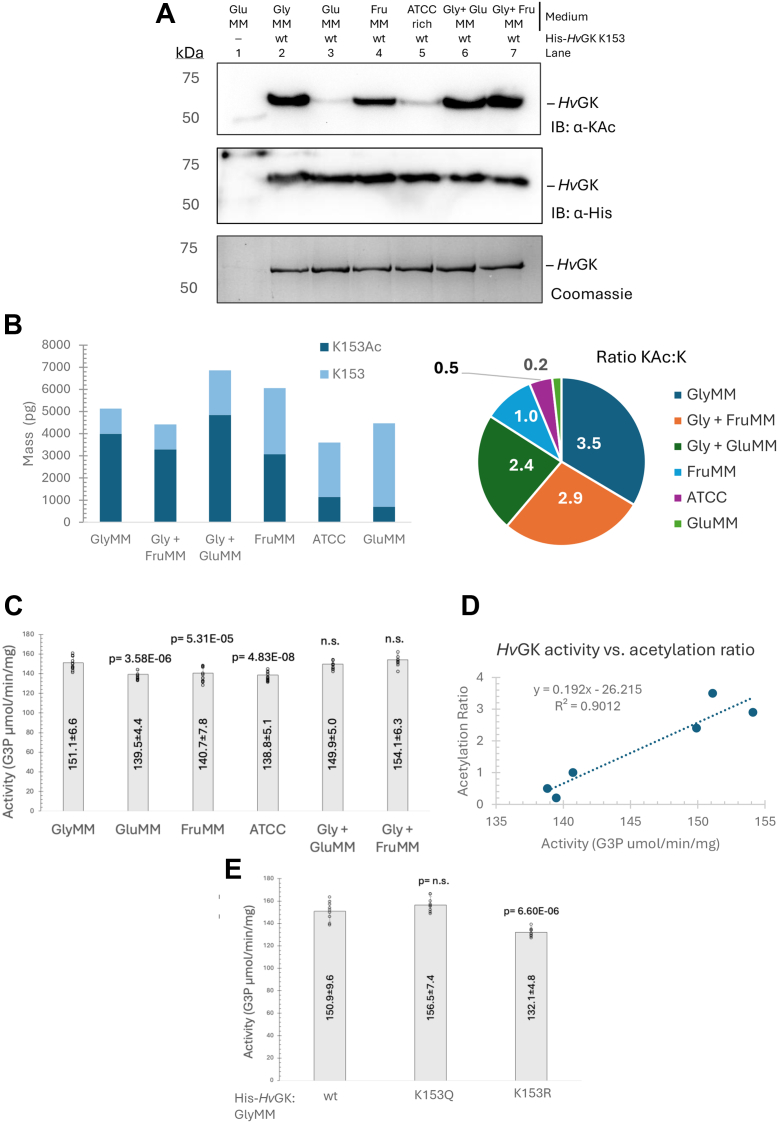
**K153-acetylated *Hv*GK abundance and enzymatic activity are positively correlated and vary across different carbon sources**. *A*, lysine acetylation status of His-*Hv*GK purified from cells grown in different carbon sources. His-*Hv*GK was purified by Ni-NTA resin from KM01-pJAM4351 cells grown to stationary phase in GlyMM, FruMM, GluMM, rich medium (ATCC), or mixed conditions (Gly + GluMM, Gly + FruMM). His-*Hv*GK proteins were separated by SDS-PAGE and detected by CB staining and IB: α-His. Lysine acetylation levels of the His-*Hv*GK proteins were analyzed by IB: α-KAc. *B*, quantification of K153 acetylation occupancy by AQUA-MS. Targeted mass spectrometry was used to determine the acetylated:nonacetylated peptide ratio at K153 under each growth condition. Acetylation occupancy was highest in GlyMM (78%, 3.5:1 ratio) and decreased in mixed or nonglycerol conditions. *C*, *Hv*GK enzyme activity varies with carbon source used for cell growth. Specific activity of His-*Hv*GK purified from cells grown on the different carbon sources was measured in a coupled assay. The *p*-values represent comparison to the His-*Hv*GK enzyme purified from GlyMM. *D*, correlation between acetylation levels and *Hv*GK activity. *Hv*GK activity (G3P μmol/min/mg) was plotted against K153 acetylation ratios from AQUA-MS analysis. A linear regression fit yielded y = 0.192x – 26.215 with R^2^ = 0.90, indicating a strong positive correlation between acetylation and enzymatic activity. *E*, activity of acetylation mimic and nonacetylated *Hv*GK variants. Specific activity of His-*Hv*GK wt, K153Q (acetylation mimic), and K153R (nonacetylated mimic) proteins purified from *H*. *volcanii* KM03 strains grown on GlyMM. The *p*-values represent comparison to His-*Hv*GK wt. Enzymatic activity values represent the mean of three independent experiments, each with three technical replicates. Statistical significance was determined using an unpaired two-tailed *t* test (*p* < 0.05 considered significant; n.s., not significant). ATCC, tryptone/yeast extract; CB, Coomassie blue; FruMM, fructose minimal medium; GK, glycerol kinase; GluMM, glucose minimal medium; *Hv*GK, *H*. *volcanii* GK; IB: α-His, immunoblotting using anti-His antibodies; IB: α-KAc, immunoblotting using anti-acetyllysine antibodies.

To investigate whether acetylation status correlates with *Hv*GK enzymatic activity, His-*Hv*GK was plasmid expressed and purified from a *ΔglpK* strain (KM01) grown in different carbon sources, including glycerol [glycerol minimal medium (GlyMM)], fructose [fructose minimal medium (FruMM)], glucose [glucose minimal medium (GluMM)], and ATCC rich media. Additionally, His-*Hv*GK was analyzed from cells grown in mixed carbon sources (GlyMM + GluMM and GlyMM + FruMM). Purified His-*Hv*GK proteins were evaluated by SDS-PAGE, IB (α-His and α-KAc), targeted quantitative MS (AQUA analysis), and GK activity assays. SDS-PAGE and IB revealed distinct lysine acetylation patterns depending on the carbon source, with the highest levels of acetylated His-*Hv*GK detected in cells grown with glycerol supplementation (Fig. 6*A*). AQUA-MS analysis demonstrated that K153-acetylated *Hv*GK levels were highest in glycerol-grown samples, with the ratio of acetylated to nonacetylated peptide reaching 3.5 in GlyMM, revealing 78% occupancy of the K153 acetylation site. Acetylation ratios decreased progressively under mixed or alternative carbon sources: 2.9 (74%) in Gly + FruMM, 2.4 (71%) in Gly + GluMM, 1.0 (50%) in FruMM, 0.5 (33%) in ATCC rich medium, and 0.2 (17%) in GluMM (Fig. 6*B*). *Hv*GK purified from glycerol-grown cells, which showed the highest acetylation, also displayed the highest specific enzymatic activity (Fig. 6*C*). Plotting activity against acetylation ratio revealed a strong positive correlation (R^2^ = 0.90), indicating that higher acetylation levels are associated with increased *Hv*GK activity (Fig. 6*D*). This correlation suggests that acetylation enhances *Hv*GK catalytic efficiency. Additionally, the acetylation mimic variant K153Q exhibited enzymatic activity comparable to wt His-*Hv*GK, whereas the nonacetylated mimic K153R displayed reduced activity relative to wt and K153Q (Fig. 6*E*). Together, these results suggest that acetylation at K153 enhances His-*Hv*GK activity and is responsive to carbon source availability.

### Acetylation state influences H*v*GK oligomeric conformation

**Figure 7:**
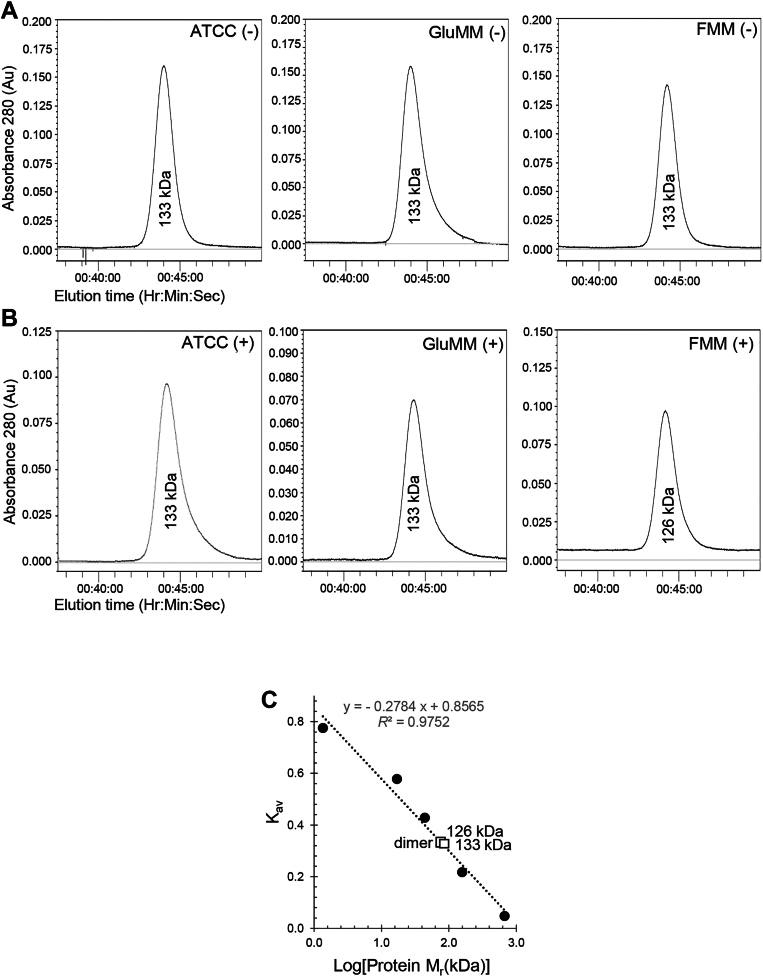
***Hv*GK oligomeric state under different carbon source and buffer conditions**. *A*, His-*Hv*GK purified from *H*. *volcanii* cells grown in ATCC, GluMM, or FruMM elutes as a dimer. Size-exclusion chromatography (SEC) profiles of His-*Hv*GK purified from *H*. *volcanii* grown in ATCC rich medium, GluMM, or FruMM show a single predominant peak corresponding to a homodimer (∼113 kDa). No peaks corresponding to tetrameric forms were observed under these growth conditions. *B*, glycerol supplementation during chromatography does not alter oligomeric state. His-*Hv*GK purified from the same conditions as in *panel A* were analyzed by SEC in buffer supplemented with 10% (v/v) glycerol. Elution profiles remained consistent with the dimeric state, indicating that addition of glycerol during chromatography does not promote higher-order assembly. *C*, SEC standard curve confirms dimeric His-*Hv*GK elution profile. SEC elution positions of His-*Hv*GK were compared to molecular mass standards (*closed circles*) using Kav (Mr) values. His-*Hv*GK homodimer (*open square*) eluted at Kav 0.272 (126 kDa) and 0.265 (133 kDa), consistent with a dimeric state. Theoretical molecular weights are as follows: 56.7 kDa monomer and 113.4 kDa dimer. SEC was performed using Superdex 200 HR 10/300 Gl columns in 50 mM HEPES (pH 7.5), 2 M NaCl, 1 mM DTT, with or without 10% glycerol. ATCC, tryptone/yeast extract; DTT, dithiothreitol; FruMM, fructose minimal medium; GK, glycerol kinase; GluMM, glucose minimal medium; *Hv*GK, *H*. *volcanii* GK.

Our previous work showed that *Hv*GK purified from glycerol-grown cells predominantly exists as a homodimer but shifts to a homotetramer when glycerol is included in the size-exclusion chromatography (SEC) buffer (3). To further evaluate how oligomerization correlates with acetylation status and carbon source, we performed SEC analyses of *Hv*GK purified from cells grown in GluMM, FruMM, and rich medium (ATCC). In all these nonglycerol conditions, His-*Hv*GK eluted as a dimer, even when 10% glycerol was added during chromatography (Fig. 7, *A*–*C*). Only *Hv*GK purified from glycerol-grown cells and analyzed in the presence of glycerol in the SEC buffer displayed a tetrameric population, as reported previously (3). These findings indicate that acetylation associated with growth on glycerol enables—but does not alone drive—the tetrameric transition, which additionally requires glycerol as a ligand during chromatography.

### Impact of K153 acetylation and ligand binding on H*v*GK thermal stability

Thermal shift assays were performed to assess how acetylation at K153 affects the thermal stability of His-*Hv*GK. The thermostability of the His-*Hv*GK wt protein was compared to the two variants: K153Q and K153R (Table 1). In the absence of ligands, both the wt and K153Q variant proteins exhibited a melting temperature (Tm) of 79 °C, whereas the K153R variant showed reduced stability, with a Tm of 77 °C. In the presence of ATP, the K153R variant again had a lower thermal stability with a Tm of 78 °C when compared to the wt and K153Q enzymes which displayed a Tm of 80 °C. Similar patterns were observed in the presence of MgCl_2_, ATP plus glycerol, ATP plus MgCl_2_, and the combination of all three ligands, where K153R variant consistently showed significantly lower Tm values than wt. Moreover, the K153Q variant mirrored or slightly exceeded wt thermal stability across the tested conditions. The exception was glycerol alone, which increased the Tm of all three enzymes to 85 to 86 °C, with no significant differences observed under this condition. Overall, these results indicate that acetylation at K153 enhances His-*Hv*GK thermal stability and that glycerol binding provides a substantial stabilizing effect regardless of acetylation status, likely contributing to enzyme resilience under metabolically relevant conditions.

**Table 1:** Melting temperature (Tm) values as determined by differential scanning fluorimetry (DSF)

*H*. *volcanii* strainvolcanii strain	KM03-pJAM4351	KM03-pJAM4354	KM03-pJAM4355
His-*Hv*GK:	Wt	K153Q	K153R
KAc state:	High Ac	Mimic Ac	Mimic no Ac
Ligand(s)	Tm values (^⸰^C)^a^		
ATP + Mg + Glycerol	90 ± 0.5	90 ± 0.6 (n.s.)	88 ± 0.0^b^
ATP	80 ± 0.5	80 ± 0.0 (n.s.)	78 ± 0.6^b^
Mg	78 ± 1.0	78 ± 0.0 (n.s.)	76 ± 0.6^b^
Glycerol	85 ± 0.0	86 ± 0.6 (n.s.)	86 ± 0.0 (n.s.)
ATP + Glycerol	88 ± 0.6	88 ± 0.6 (n.s.)	86 ± 0.0^b^
Mg + Glycerol	84 ± 0.0	84 ± 0.0 (n.s.)	85 ± 0.0 (n.s.)
ATP + Mg	81 ± 0.6	81 ± 0.6 (n.s.)	79 ± 0.6^b^
Buffer only	79 ± 0.5	79 ± 0.6 (n.s.)	77 ± 0.6^b^

a Tm values of His-*Hv*GK wt, K153Q, and K153R enzymes were determined by DSF following purification by Ni-NTA affinity chromatography from *H*. *volcanii* KM03 strains grown to stationary phase in GlyMM. Assays were performed in 50 mM HEPES (pH 7.5), 2 M NaCl, and included ATP (35 mM), MgCl_2_ (35 mM), and glycerol (46 mM) as indicated. The wt His-*Hv*GK (pJAM4351) was used as the reference for statistical comparisons against K153Q (acetylation mimic; pJAM4354) and K153R (nonacetylated mimic; pJAM4355). Statistical analysis was performed using unpaired two-tailed *t* tests. Significant differences (*p* < 0.05) between wt and K153R were observed under the following conditions: no ligands (*p* = 2.63 × 10^-3^), ATP (*p* = 4.09 × 10^-4^), MgCl_2_ (*p* = 0.01), ATP + glycerol (*p* = 3.54 × 10^-3^), ATP + MgCl_2_ (*p* = 4.42 × 10^-3^), and ATP + MgCl_2_ + glycerol (*p* = 4.14 × 10^-3^). No significant differences were detected between wt and K153Q under any condition.

b In the table indicates a significant difference relative to wt; “n.s.” = not significant. All measurements were performed in biological triplicate. Supporting data are provided in Figs. S2–S4.

### Acetylation at K153 modulates H*v*GK kinetics and allosteric behavior

**Figure 8:**
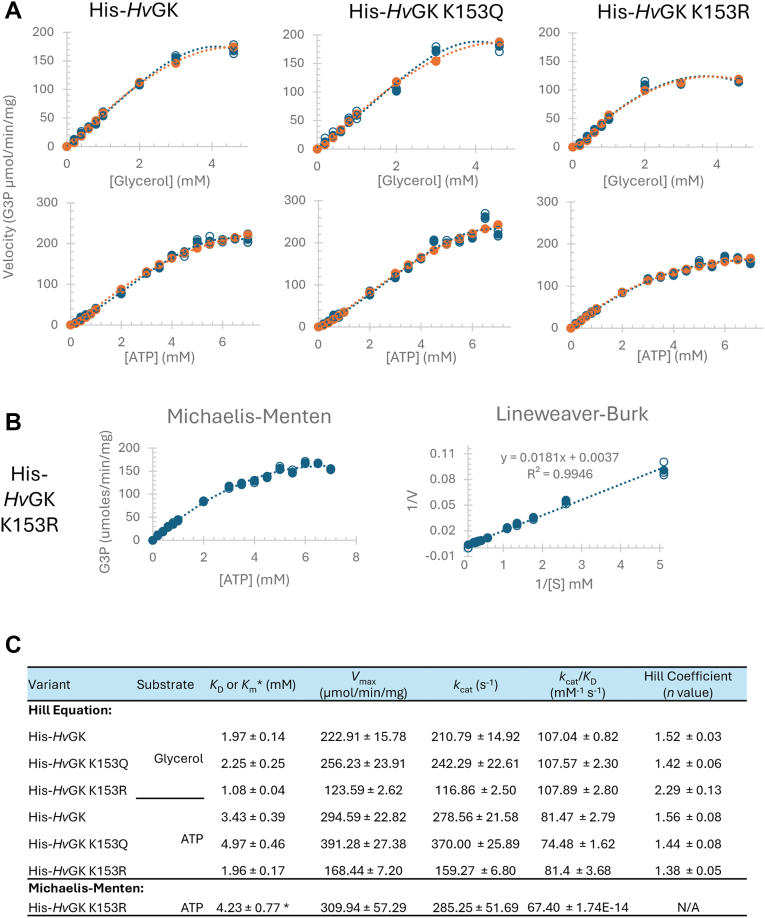
**Kinetic analysis of *Hv*GK variants purified from *H*. *volcanii* KM03 grown in GlyMM**. *A*, enzymatic activity of His-*Hv*GK wt, K153Q (acetylation mimic), and K153R (nonacetylatable mutant) was measured under optimal assay conditions (50 mM HEPES, pH 8.0, 100 mM NaCl, 57 °C) using a coupled spectrophotometric assay that monitors NADH oxidation at 340 nm. Substrate concentrations were varied using glycerol and ATP. The kinetic profiles of His-*Hv*GK wt and K153Q exhibited sigmoidal (non-Michaelis–Menten) behavior for both glycerol and ATP, consistent with cooperative substrate binding. In contrast, K153R showed Michaelis–Menten-like kinetics with ATP, indicating a loss of allosteric behavior. Experimental velocity data (*blue*) and best-fit model values (*orange*) are shown for each condition. *B*, Michaelis–Menten and Lineweaver–Burk plots for the K153R variant with ATP further support the noncooperative kinetic behavior of this mutant. *C*, summary of kinetic parameters: Km, KD, Vmax, kcat, kcat/KD, and Hill coefficient (n) for each enzyme–substrate combination. Data were fit using the Hill or Michaelis–Menten equations as appropriate. The Michaelis–Menten equation describes a simple enzyme–substrate interaction as *v* = (*V*_max_ [S])/(*K*_m_ + [S]), where *v* is the reaction rate, *V*_max_ is the maximum rate, [S] is the substrate concentration, and *K*_m_ is the Michaelis constant. Hill equation used was calculated Y = (*V*_max_)[L]^ˆ^*n*/(*K*_D_ + [L]^ˆ^*n*), where Y represents the fraction of occupied binding sites, [L] is the ligand concentration, *n* is the Hill coefficient indicating cooperativity, and *K*_D_ is the dissociation constant. Squared residuals (Yexperimental − Ycalculated)^2^ were summed to compute the sum of square residuals (SSRs). Excel Solver was employed to minimize SSR and optimize the parameters for the best fit of the data. All measurements represent the mean of three biological replicates. GK, glycerol kinase; GlyMM, glycerol minimal medium; *Hv*GK, *H*. *volcanii* GK.

To assess how K153 acetylation influences *Hv*GK activity and substrate recognition, kinetic parameters were determined for the wt, K153Q, and K153R enzymes. All enzymes were purified with a His-tag from a *ΔglpK* background (KM03) to ensure homogeneity. The assays were performed under optimal activity conditions. With glycerol as the variable substrate, both His-*Hv*GK wt and K153Q variant displayed similar *K*_D_ values (∼2 mM) and high catalytic turnover (*k*_cat_ > 210 s^-1^), with Hill coefficients of 1.52 and 1.42, respectively, indicating positive cooperativity (Fig. 8, *A* and *C*). In contrast, K153R showed a nearly 2-fold reduction in *k*_cat_ (∼117 s^-1^), despite a lower apparent *K*_D_ (1.08 mM), suggesting loss of catalytic efficiency. Interestingly, the K153R variant exhibited an even higher Hill coefficient (*n* = 2.29), pointing to altered cooperative behavior possibly arising from conformational or oligomeric shifts (Fig. 8*C*). When ATP was varied, the His-*Hv*GK wt and K153Q variant enzymes retained sigmoidal kinetics, with Hill coefficients ∼1.5 and *k*_cat_ values exceeding 270 s^-1^. In contrast, the K153R variant displayed a noticeably more hyperbolic profile. When fit to the Hill equation, the K153R variant showed a modest Hill coefficient (*n* = 1.38), but the kinetic curve was more accurately described by the Michaelis–Menten model, with a *K*_m_ of 4.23 mM (Fig. 8, *B* and *C*). This attenuation of sigmoidal behavior indicates reduced cooperativity toward ATP and supports that acetylation at K153 contributes to maintaining an allosterically responsive conformation.

### Pat2-mediated acetylation of H*v*GK K153 appears to alter growth on glycerol

**Figure 9:**
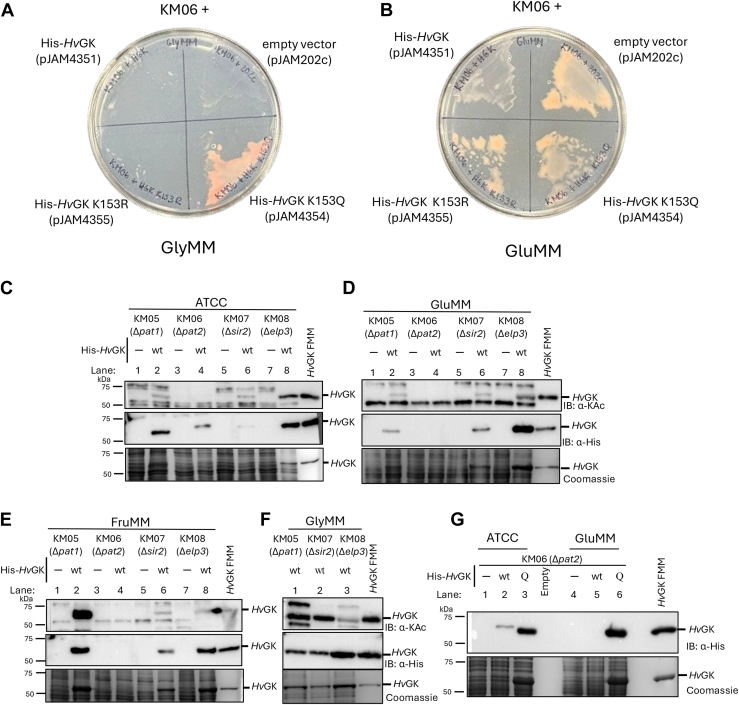
**Pat2 acetylation or the K to Q acetylation mimic of the K153 site are associated with *Hv*GK protein abundance and *H*. *volcanii* growth on glycerol**. *A*, *Δpat2ΔglpK* mutant strains show growth defects on GlyMM when lacking *Hv*GK (empty vector) or expressing His-*Hv*GK wt or K153R from a plasmid, compared to the K153Q variant. KM06 (*ΔglpKΔlarCΔpat2*) strains expressing His-*Hv*GK wt, K153Q, K153R, or empty vector were streaked with loop onto GlyMM plates and incubated for 8 days. Growth was observed for the KM06 strain expressed the *Hv*GK K153Q acetylation mimic compared to the other strains. *B*, robust growth on GluMM observed for all *Δpat2 ΔglpK* mutant strains examined irrespective of the presence or absence of *Hv*GK wt or variants. KM06 strains (as in *panel A*) were streaked onto on GluMM and incubated for 8 days. Growth was monitored by colony/streak formation. *C–G*, differential effects of *Δpat2*, *Δsir2*, and K153Q substitution on *Hv*GK protein abundance and lysine acetylation across carbon sources, relative to *Δpat1* and *Δelp3* strains. KM05 *(Δpat1*), KM06 *(Δpat2*), KM07 (*Δsir2*), and KM08 (*Δelp3*) strains expressing His-*Hv*GK from plasmid pJAM4351 (wt) and His-*Hv*GK K153Q from plasmid pJAM4354 (Q) compared to the empty vector control pJAM202c (−). The GNAT family (*pat1*, *pat2*, and *elp3*) and Sir2-type deacetylase (*sir2*) gene homolog mutations were generated in parent strain KM03 (H1207 *ΔglpK ΔlarC*). Cells were grown in carbon sources, including ATCC 974 rich, GluMM, FruMM, and GlyMM, as indicated. Whole cells were normalized to 0.1 OD_600_ per lane and lysed by boiling for 10 min in Laemmli SDS sample buffer. Proteins were separated by SDS-PAGE. Equal protein loading was confirmed by CB stain. His-*Hv*GK abundance and lysine acetylation were detected by IB: α-His and IB: α-KAc, respectively. His-*Hv*GK purified from KM01 grown in FruMM was included as a positive control where indicated (*Hv*GK FMM). KM06 and empty vector strains failed to grow in GlyMM, preventing protein analysis under these conditions. See Materials and Methods for details. All experiments were performed in biological triplicates. Representative immunoblots are shown. ATCC, tryptone/yeast extract; CB, Coomassie blue; FruMM, fructose minimal medium; GK, glycerol kinase; GluMM, glucose minimal medium; GlyMM, glycerol minimal medium; *Hv*GK, *H*. *volcanii* GK; IB: α-His, immunoblotting using anti-His antibodies; IB: α-KAc, immunoblotting using anti-acetyllysine antibodies.

Our recent work demonstrates that Pat2 acetylates *Hv*GK (27); however, the functional consequences of this modification remained unclear. To address this, we assessed the ability of His-*Hv*GK or its variants to complement growth of a *ΔglpK* mutant in a *Δpat2* background (KM06). Growth plate assays demonstrated that expression of either His-*Hv*GK wt or the nonacetylated mimic K153R failed to rescue growth on GlyMM, whereas the K153Q acetylation mimic fully restored growth of the KM06 mutant under these conditions (Fig. 9*A*). All constructs displayed growth on GluMM, confirming that the growth defect is specific to glycerol metabolism (Fig. 9*B*). These findings highlight a previously unrecognized requirement for the Pat2-mediated K153 acetylation in enabling *Hv*GK function under glycerol-utilizing conditions that is not needed for growth on glucose.

### Acetylation of H*v*GK K153 impacts H*v*GK abundance on glycerol

To further investigate the molecular basis of this phenotype, His-*Hv*GK abundance and lysine acetylation status were determined in strains with GNAT-family acetyltransferase mutations *Δpat1* (KM05), *Δpat2* (KM06), and *Δelp3* (KM08) and the Sir2-type deacetylase mutation *Δsir2* (KM07). The strains, which were also *ΔglpK* mutants, were transformed with plasmids expressing from a P2 promoter either His-*Hv*GK wt or the empty vector to enable controlled assessment of *Hv*GK. The transformed cells were cultured on various carbon sources, and their lysates were analyzed by SDS-PAGE and IB using α-His to assess *Hv*GK abundance and α-KAc to evaluate lysine acetylation. Bands corresponding to lysine-acetylated wt His-*Hv*GK in the cell lysates were identified by comparison with empty vector controls and comigration with a purified, lysine-acetylated His-*Hv*GK standard. By this strategy, His-*Hv*GK was detected and found lysine acetylated in KM05 (*Δpat1*) across all carbon sources (Fig. 9, *C*–*E lane 2* and Fig. 9*F lane 1*), with the highest acetylation levels observed in FruMM and GlyMM, indicating that *pat1* is not essential for *Hv*GK acetylation. While fluctuations in *Hv*GK abundance and lysine acetylation were also observed across different carbon sources in the KM07 (*Δsir2*) and KM08 (*Δelp3*) strains, *Hv*GK was still detected and found lysine acetylated in these strains (Fig. 9, *C*–*E*, *lane 6 and 8*, Fig. 9*F lane 2 and 3*). In contrast, the lysine acetylated form of His-*Hv*GK was not detected in KM06 (*Δpat2*) under any of the tested carbon sources (Fig. 9, *C*–*E*, *lane 4*). Additionally, His-*Hv*GK abundance was severely reduced in this strain: undetectable in GluMM and FruMM (Fig. 9, *D* and *E*, *lane 4*) and of limited abundance in ATCC medium (Fig. 9*C*, *lane 4*). This reduction in His-*Hv*GK abundance is consistent with a loss of lysine acetylation-dependent stabilization of the protein, as Pat2 is the primary acetyltransferase responsible for modifying K153. Although the K153R variant demonstrates that *Hv*GK can still fold and retain residual activity without acetylation (Fig. 6*E*), its decreased stability and cooperativity support the interpretation that acetylation of lysine 153 promotes enzyme persistence and functional efficiency *in vivo*. Accordingly, the *Δpat2* mutation significantly lowers *Hv*GK abundance, and when combined with the allosteric and stability defects of the nonacetylatable K153R variant, glycerol utilization falls below the flux threshold required for growth (Fig. 9).

The ability to restore growth of the KM06 strain (*ΔglpK ΔlarC Δpat2*) on GlyMM by expressing His-*Hv*GK K153Q *versus* wt or K153R, together with our other findings, suggested that Pat2-mediated acetylation at K153 plays a role in supporting growth on glycerol and may be positively associated with *Hv*GK protein abundance. To further investigate this possibility, the protein abundance of the His-*Hv*GK K153Q variant was analyzed in parallel with the wt His-*Hv*GK in the KM06 strain grown on GluMM and ATCC media. The K153Q variant was markedly more abundant than wt *Hv*GK in both media types to the extent that it was detectable by both α-His IB and CB staining of the cell lysate (Fig. 9*G*). This finding suggests that mimicking acetylation at K153 is sufficient to bypass the requirement for Pat2 in maintaining *Hv*GK abundance. Together these results highlight the specific and critical role of Pat2 in the K153 acetylation of *Hv*GK and reveal the positive impact this PTM has on the abundance of *Hv*GK in central metabolism.

## Discussion

Lysine acetylation and other PTMs are well-established regulators of metabolic enzymes in bacteria and eukaryotes, and this mechanism is now demonstrated to also play a key role in controlling central carbon metabolism in archaea. Specifically, Pat2-mediated acetylation of a single lysine residue (K153) on *Hv*GK enhances the activity, stability, and abundance of this enzyme on glycerol relative to glucose, the less-preferred carbon source, in *H*. *volcanii*. This K153 acetylation of *Hv*GK reflects a metabolic prioritization strategy that directs glycerol into glycerol-3-phosphate, feeding pathways to pyruvate and acetyl-CoA to support energy generation and cellular carbon biosynthesis.

The acetylation site K153 on *Hv*GK resides within a conserved flexible loop, suggesting a mechanism for regulating enzyme conformational dynamics that is evolutionarily maintained across haloarchaea (*Halobacteria*). This modification likely enables haloarchaea to efficiently metabolize glycerol released from lysed or leaking cells in hypersaline environments, allowing rapid utilization of available carbon sources and supporting metabolic flexibility under nutrient-limited conditions. Flexible loops are common targets of PTMs, often modulating enzyme activity through conformational changes (28, 29, 30, 31). Our findings extend this regulatory paradigm to archaeal enzymes and suggest a link between PTM-mediated control of enzyme dynamics and ecological adaptation.

Pat2 directly acetylates *Hv*GK lysine 153, establishing a mechanistic link between this GNAT family acetyltransferase and *Hv*GK regulation. These findings parallel well-characterized site-specific lysine acetylation events that modulate protein function, including modification of the AMP-forming acetyl-CoA synthetase in bacteria (32) and Alba in crenarchaea (33, 34), underscoring acetylation of structurally critical lysine residues is an ancient and conserved regulatory mechanism. In the absence of *pat2*, *Hv*GK lysine acetylation and abundance are substantially reduced but can be overcome by mimicking acetylation with a K153Q substitution. Thermal shift analyses further demonstrate that acetylation at K153 enhances structural stability, especially in the presence of substrates. Notably, wt *Hv*GK purified from glycerol-grown cells—where lysine 153 is highly acetylated—showed growth and kinetic behavior nearly identical to the K153Q variant. This parallel supports that K153Q mimics the acetylated enzyme and that its enhanced stability arises from charge neutralization rather than side-chain–specific effects. These results indicate that acetylation not only regulates activity but also protects *Hv*GK from degradation, paralleling acetylation-driven stabilization mechanisms observed in eukaryotic kinases such as HIPK2 (35), LATS1 (36), and GSK-3β (37).

*Hv*GK purified from glycerol-grown cells undergoes a glycerol-dependent dimer-to-tetramer transition, a shift not observed in *Hv*GK purified from cells grown on alternative carbon sources associated with lower acetylation occupancy. This correlation suggests that acetylation enhances the enzyme’s capacity to adopt higher-order oligomeric states, but glycerol binding is required to stabilize the tetrameric conformation. Consistent with this model, the acetyl-mimic variant K153Q retains positive cooperativity with both glycerol and ATP, whereas the nonacetylable K153R displays loss of allosteric regulation and reduced thermostability, particularly in the presence of ATP. The transition from cooperative to noncooperative kinetics in these variants underscores the role of K153 acetylation in maintaining an allosterically competent and structurally flexible enzyme conformation. Structural modeling further supports this interpretation: in the dimeric model of *Hv*GK, K153 is positioned on a solvent-exposed loop distant from both active sites and the dimer interface but proximal to the interdimer contact observed in low-confidence tetramer predictions. This spatial arrangement suggests that acetylation at K153 could modulate interdimer interactions and promote stabilization of the tetrameric state under high-acetylation, glycerol-rich conditions, thereby coupling structural assembly with enzymatic regulation.

Together, these results support a threshold model for HvGK regulation: Pat2-dependent acetylation maintains enzyme abundance and stability, while K153 acetylation (or charge-neutral substitution) preserves allosteric competence. Substitution with K153R reduces glycerol flux yet still permits limited growth. However, when both factors are absent (*Δpat2* combined with K153R), glycerol utilization falls below the metabolic threshold required to sustain growth, resulting in a synthetic-sick phenotype. This framework reconciles the measurable activity and partial complementation of K153R with its inability to support growth in the *Δpat2* background, highlighting the interplay between enzyme dosage, allosteric regulation, and metabolic flux control *in vivo*.

**Figure 10:**
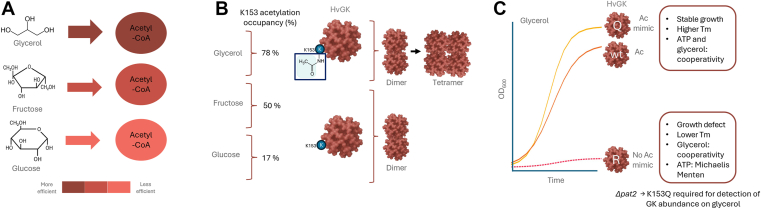
**Working model: Acetylation of *Hv*GK regulates glycerol metabolism, protein abundance, and allosteric control in *H*. *volcanii***. *A*, glycerol metabolism yields abundant acetyl-CoA through a streamlined pathway involving glycerol kinase (*Hv*GK) providing higher energetic efficiency than glucose or fructose utilization. *B*, *Hv*GK is acetylated at lysine 153 (K153) in a carbon source–dependent manner, with the highest acetylation level (78%) observed during. Growth on glycerol. Elevated acetylation correlates with the enzyme’s ability to undergo a glycerol-dependent dimer-to-tetramer transition, whereas *Hv*GK from other carbon sources remains dimeric. *C*, functional comparison of *Hv*GK variants demonstrates that K153 acetylation enhances protein abundance, stability, and allosteric regulation. Both wt *Hv*GK and the acetylation-mimetic K153Q (Q) variant support growth in glycerol, show high structural stability, and maintain cooperative binding for both glycerol and ATP. In contrast, the nonacetylatable K153R (R) variant exhibits poor growth in glycerol, reduced thermal stability, and a loss of allosteric regulation for ATP (Michaelis–Menten kinetics), while retaining cooperativity for glycerol. Deletion of the acetyltransferase Pat2 strongly reduces *Hv*GK abundance, whereas the Q variant remains stable, underscoring acetylation’s dual role in maintaining protein levels and regulating metabolic adaptation. GK, glycerol kinase; *Hv*GK, *H*. *volcanii* GK.

Overall, our findings support a model (Fig. 10, *A*–*C*) in which K153 acetylation regulates *Hv*GK oligomeric state, activity, stability, and abundance, establishing *Hv*GK as a dynamic regulatory node in central carbon metabolism. This model provides insight into the regulatory mechanisms used by *H*. *volcanii* to favor the metabolism of glycerol over glucose and the coutilization of glycerol and fructose (7, 8, 38). By stabilizing and promoting adaptive oligomerization and activity of *Hv*GK, the K153 acetylation ensures metabolic flexibility. The 78% occupancy of the K153 acetylation site on *Hv*GK during growth on glycerol suggests that this abundant metabolic enzyme may act as a reservoir of acetyl groups, with deacetylation potentially replenishing acetyl-CoA pools or supporting energy-conserving acetyltransferase reactions under energy- or carbon-limited conditions, as proposed for histone deacetylation (39). Given that glycerol has industrial relevance as a major biodiesel byproduct (40, 41, 42), understanding acetylation-based regulation provides a foundation for engineering archaeal enzymes for sustainable biocatalysis. The acetyl-mimic K153Q variant, which combines stability and enhanced activity, offers a promising starting point for future metabolic engineering strategies to control key metabolic steps like glycerol phosphorylation (43, 44).

## Experimental procedures

### Strains and media

Strains, plasmids, and oligonucleotides used in this study are listed in Tables S1-S2. *E*. *coli* Top10 (Invitrogen) was used for routine cloning, and *E*. *coli* GM2163 (New England Biolabs) was used for propagation of unmethylated plasmids for *H*. *volcanii* transformations. *E*. *coli* strains were grown at 37 °C in Luria-Bertani medium supplemented with 100 μg/ml ampicillin and/or 34 μg/ml chloramphenicol as needed. *H*. *volcanii* strains were cultured at 42 °C in ATCC 974 complex medium (ATCC) or minimal media supplemented with glycerol (GlyMM), glucose (GluMM), or fructose (FruMM) at 20 mM each carbon source final concentration. Growth media were supplemented as needed with uracil (50 μg/ml), novobiocin (Nov, 0.1 μg/ml), 5-fluoroorotic acid (50 μg/ml dissolved in DMSO), and isopropyl-β-D-1-thiogalactopyranoside (0.4 mM). For growth assays, colonies from solid media were inoculated into 5 ml of medium. Cells were subcultured at an initial optical density monitored at 600 nm (OD_600_) of 0.02. Cell growth was monitored by an increase in OD_600_, where 1 OD_600_ unit equals 1 × 10^9^ colony forming units (CFU)·ml^-1^ for all strains used in this study.

### Construction of *H*. *volcanii* strains

Markerless gene deletions were generated using the *pyrE2*-based "pop-in/pop-out" method as previously described (45). Flanking regions (∼500 bp) were amplified and cloned into the BamHI and HindIII sites of pTA131. The resulting plasmids were transformed into *H*. *volcanii* strains that included the *ΔpyrE2* mutation, and transformants were selected on uracil-deficient media (*Hv*-CA+). Counter-selection was performed on 5-fluoroorotic acid–containing plates (ATCC). Mutants were identified by PCR screening and confirmed by Sanger sequencing using primers outside of the 500 bp flanking regions. *H*. *volcanii* H1207 (*ΔpyrE2* *ΔpitA)* was the parental strain used to generate the double mutant strain H1207 *ΔglpK* *ΔlarC* (designated KM03). KM03 was constructed to eliminate co-purification of the endogenous His-rich LarC (HVO_2381, putative nickel insertion protein) (Fig. S1). Additional strains derived from KM03, including *Δpat1* (KM05), *Δpat2* (KM06), *Δsir2* (KM07), and *Δelp3* (KM08), were generated to investigate the effects of acetyltransferase and deacetylase mutations on *Hv*GK regulation *in vivo*.

### Plasmid construction

The plasmids used in this study are listed in Table S2. High-fidelity PCR was used to amplify the *H*. *volcanii glpK* gene (*hvo_1541*) using Phusion DNA polymerase (New England Biolabs) and cloned into two different expression systems. For native expression in *H*. *volcanii*, the *glpK* gene was inserted into the pJAM503 backbone modified for N-terminal His-tag fusion, generating plasmid pJAM4351. For recombinant expression in *E*. *coli* Rosetta (DE3), the gene was cloned into the pET15b vector, generating plasmid pJAM4360. Amplified inserts and vectors were digested with NdeI and BlpI restriction enzymes, purified using the Monarch PCR and DNA Cleanup Kit (New England Biolabs), ligated with T4 DNA ligase, and transformed into *E*. *coli* Top10 cells. Positive clones were screened by colony PCR and verified by Sanger DNA sequencing (Eurofins Genomics). Site-directed mutagenesis to generate the K153R and K153Q variants of *Hv*GK was performed by the SSPER and rrPCR methods previously described (46), followed by DpnI digestion and transformation into *E*. *coli* GM2163 for plasmid propagation. Verified plasmids were subsequently transformed into *H*. *volcanii* H1207 for native expression or *E*. *coli* Rosetta (DE3) for recombinant expression.

### Protein modeling and multiple sequence alignment

The predicted 3D structures of proteins were generated using AlphaFold3 and AlphaFold2 (47) *via* the ColabFold implementation (48), as indicated. Structural visualization and analysis were conducted using UCSF ChimeraX v1.5 (49). Confidence in the models was assessed using pTM/ipTM scores. Oligomeric models of *Hv*GK were compared with experimentally determined GK dimer structures (PDB IDs: 6ZQ7, 6K78, and 6J9X) using the ChimeraX matchmaker tool. This tool was also used to determine sequence alignment scores and calculate RMSD values over pruned Cα pairs and across all matched pairs. Multiple sequence alignment of *Hv*GK homologs (Dataset S1) was performed using Clustal Omega (50). Alignment results were visualized using SnapGene v. 8.0.2 software from Dotmatics.

### H*v*GK expression and purification

His-tagged *Hv*GK expression and purification from *H. volcanii* were carried out as previously described (3). Briefly, *H*. *volcanii* strains were grown to stationary phase, harvested, and lysed in buffer containing 50 mM HEPES (pH 7.5), 2 M NaCl, 5 mM β-mercaptoethanol, 40 mM imidazole, DNase I, and protease inhibitors. Lysates were clarified by centrifugation and incubated with Ni-NTA resin (Millipore Sigma) at 4 °C. Proteins were eluted with 250 mM imidazole in HEPES buffer and analyzed by SDS-PAGE. For *Hv*GK_Ec_, *E*. *coli* Rosetta (DE3) cells carrying pJAM4360 were induced with 0.4 mM isopropyl-β-D-1-thiogalactopyranoside at 25 °C overnight as previously descirbed (27). *Hv*GK_Ec_ was purified similarly, with buffer exchange to 2 M NaCl using Zeba spin desalting columns to enhance protein stability (27).

### Mapping acetylation sites and protein identification by LC-MS/MS analysis

The following strategy was used for mapping acetylation sites and identifying unknown proteins. Protein samples were purified by Ni-NTA affinity chromatography, separated by SDS-PAGE, stained with BioSafe CB, and excised from the gel prior to LC-MS/MS analysis. Samples included *Hv*GK expressed from a plasmid with an N-terminal His-tag and purified from *H*. *volcanii* H1207-pJAM4351 cells grown in GlyMM. An additional protein that copurified from glucose- (GluMM-) grown *H*. *volcanii* H1207 cells carrying the empty vector (pJAM202c) was also analyzed. For comparison, recombinant His-*Hv*GK was analyzed following heterologous expression from plasmid pJAM4360 in *E*. *coli* Rosetta (DE3) cells, grown in Luria–Bertani medium supplemented with chloramphenicol.

Excised protein bands were subjected to in-gel digestion using sequencing-grade trypsin or chymotrypsin (Promega), following the manufacturer’s protocol with minor modifications. Gel bands were trimmed closely to reduce background and washed twice with nanopure water, followed by two 10-min washes with 1:1 (v/v) methanol: 50 mM ammonium bicarbonate. Dehydration was performed with 1:1 (v/v) acetonitrile: 50 mM ammonium bicarbonate. Gel slices were reduced with 25 mM dithiothreitol (DTT) in 100 mM ammonium bicarbonate for 30 min and alkylated with 55 mM iodoacetamide for 30 min in the dark. Following another round of washing and dehydration, protease digestion was initiated by rehydrating gels with 12 ng/μl trypsin in 0.01% ProteaseMAX surfactant, followed by overlaying with 40 μl of 0.01% ProteaseMAX in 50 mM ammonium bicarbonate. Digestion was allowed to proceed for 1 h with gentle shaking. Reactions were quenched with 0.5% trifluoroacetic acid, and samples were either analyzed immediately or stored at −80 °C.

Peptides were analyzed by nanoLC-MS/MS using a Thermo Scientific Q Exactive HF Orbitrap mass spectrometer with an EASY-Spray nanosource and an UltiMate 3000 RSLCnano system (Thermo Scientific). Peptides were first loaded onto a PharmaFluidics mPAC C18 trapping column (10 mm) with 1% buffer B (acetonitrile + 0.1% formic acid) and desalted at a flow rate of 10 μl/min. Peptides were then eluted onto a 50 cm mPAC C18 analytical column and separated using a gradient from 1% to 20% buffer B over 60 min, then up to 45% over 10 min (total run time 90 min). The flow rate was initially set at 750 nl/min for 15 min and reduced to 300 nl/min thereafter. Column temperature was maintained at 40 °C.

MS was operated in data-dependent acquisition mode using the Top15 method. Full scans (m/z 375–1575) were acquired at 30,000 resolutions, with AGC target of 3e6 and maximum injection time of 50 ms. The most intense ions were selected for MS/MS at 15,000 resolutions with a 2e5 AGC target and 55 ms maximum injection time. HCD fragmentation was performed using stepped normalized collision energy of 28, with a 4.0 m/z isolation window. Singly charged ions and known isotopes were excluded. Dynamic exclusion was enabled (repeat count: 1; exclusion duration: 15 s). The siloxane ion at m/z 445.12003 served as an internal calibrant. A HeLa digest standard was analyzed regularly to ensure instrument performance, with a minimum acceptance threshold of 2700 protein IDs.

Raw MS/MS spectra were processed using Proteome Discoverer 3.2 (Thermo Fisher Scientific). Spectra were searched using the Sequest HT algorithm against *H*. *volcanii* Uniprot ID: UP000008243 supplemented with *E*. *coli* and common contaminant sequences. Search parameters included trypsin specificity, 10 ppm precursor ion tolerance, and 0.02 Da fragment ion tolerance. Carbamidomethylation of cysteine was set as a fixed modification; methionine oxidation, N-terminal acetylation, methionine loss (±acetylation), and lysine acetylation were included as variable modifications. Peptides were filtered for a 1% false discovery rate using Percolator. Acetylated lysine residues were manually validated, and MS2 fragment spectra were inspected to confirm site localization.

### Protein quantification and SDS-PAGE analysis

Protein concentrations were determined by Bradford assay using bovine serum albumin (Bio-Rad Laboratories) as a standard. Samples were resolved by 8% or 12% SDS–PAGE as indicated, and protein fractions were prepared for electrophoresis by mixing equal volume of Laemmli SDS sample buffer: 100 mM Tris-HCl (pH 6.8), 10% (v/v) β-mercaptoethanol, 2% (w/v) SDS, 10% (v/v) glycerol, and 0.6 mg/ml bromophenol blue. The samples were boiled for 10 min, chilled on ice for 5 min, and centrifuged at 13,000×*g* for 10 min. Precision Plus Protein Kaleidoscope molecular mass marker (Bio-Rad Laboratories) was the standard. The gels were stained with CB and imaged using an iBright Imaging System (Invitrogen) according to the manufacturer's protocol.

### Immunoblot analysis

After SDS-PAGE, proteins were transferred to PVDF membranes (0.2 μm) (Amersham) at 25 V for 7 min using the Trans-Blot Turbo Transfer System (Bio-Rad Laboratories) and Towbin buffer (as recommended by supplier). After protein transfer, the membranes were incubated with gentle rocking overnight at 4 °C in blocking buffer (see below). For His-tagged proteins, membranes were blocked in buffer composed of 5% (w/v) skim milk powder in TBST [50 mM Tris-HCl, pH 7.6, 150 mM NaCl, and 0.1% (v/v) Tween 20], and subsequently probed for 1 h at RT with HRP-conjugated 6 × His-tag mouse monoclonal antibody (Proteintech Group, Inc. Cat. No. HRP-660005) diluted 1:10,000 in blocking buffer. For lysine acetylation proteins, membranes were blocked in buffer composed of 5% (w/v) BSA in TBST and subsequently probed for 1 h at RT with anti-acetyllysine rabbit mAb (PTM Bio, #PTM-105RM) as the primary antibody at 1:5000 dilution and mouse anti-rabbit IgG-HRP (Santa Cruz Biotechnology; #sc-2357) as the secondary antibody at 1:10,000 dilution. Following each antibody incubation time, the membrane was washed five times with TBST for 5 min per wash. The Amersham ECL Prime substrate mixture (1:1) was applied to the membrane and incubated in the dark at RT for 5 min before imaging with the iBright Imaging System (Invitrogen) according to the manufacturer's protocol.

### Lysine acetylation assays

Lysine acetylation assays were performed, as previously described (27). Reactions (50 μl) contained 6 μM HvPat2 enzyme, 2 μM recombinant *Hv*GK_Ec_ substrate, 0.2 mM acetyl-CoA, 50 mM HEPES (pH 7.5), 2 M NaCl, and 1 mM DTT. Reactions were incubated at 37 °C for 3 h and subsequently precipitated overnight on ice by the addition of 10% (w/v) trichloroacetic acid. Samples were centrifuged (10 min, 17,999×*g*, 4 °C), and the resulting protein pellets were washed twice with 100% cold acetone. Air-dried pellets were resuspended in 20 μl 2 × SDS-PAGE reducing buffer, boiled for 5 min, and analyzed by 12% SDS-PAGE followed by CB staining and IB with anti-acetyllysine antibodies, as described above.

### Carbon shift assay

*H*. *volcanii* KM03 (*ΔglpK* *ΔlarC*) carrying plasmids encoding His-*Hv*GK ‘wt’, K153Q, and K153R enzymes, and the empty vector (pJAM202c) were used for carbon shift assays. Strains were first streaked onto GluMM plates and incubated at 42 °C. Single colonies were used to inoculate 5 ml GluMM liquid cultures, which were grown to early exponential phase. These cultures were then used to inoculate 50 ml of fresh GluMM at an initial OD_600_ of 0.02, with incubation at 42 °C under aerobic conditions.

Cells were cultured in GluMM for 4 days, with OD_600_ measurements and sample collection performed every 24 h. At each time point, 0.5 OD_600_ units of cells were harvested by centrifugation and stored at −80 °C for further analysis. On the fourth day, the remaining culture was harvested, washed three times with 18% saltwater solution (diluted from 30% stock composed per liter of 240 g NaCl, 30 g MgCl_2_^.^6H_2_O, 35 g MgSO_4_^.^7H_2_O, 7 g KCl, and 5 mL 1 M Tris-HCl, pH 7.5). Washed cells were resuspended into GlyMM at an OD_600_ of 0.02. Cells were then incubated in GlyMM for an additional 4 days, with OD_600_ measurements and sample collection as described above. Collected samples were analyzed by SDS-PAGE followed by CB staining. IB was performed with anti-His-tag antibodies to assess His-*Hv*GK protein abundance and anti-acetyllysine antibodies to evaluate lysine acetylation profiles. As a control, mock shift experiments were performed by growing cells in GluMM and then shifting to the same carbon source (GluMM).

### Viability plating of stationary phase cells

Following growth monitoring, 5 μl from each flask culture or 96-well culture were spotted onto ATCC974, FruMM, and GlyMM agar plates. Samples were also serially diluted (1:10) in GlyMM prior to spotting. Plates were incubated at 42 °C for 5 days and imaged using the iBright FL1000 Imaging System (Thermo Fisher Scientific) in universal imaging mode to assess cell viability.

### Protein preparation and trypsin digestion for AQUA-MS analysis

*Hv*GK enzymes for AQUA-MS analysis were purified from *H*. *volcanii* KM01 carrying pJAM4351 using Ni-NTA resin, as described in the previous section. Prior to purification, the cells were grown to stationary phase (*A*>1) in different carbon sources including GlyMM, GluMM, FruMM, ATCC, Gly + GluMM, and Gly + FruMM. Protein concentration was determined by Bradford assay with bovine serum albumin as the standard. Purified His-*Hv*GK (50 μg) was denatured in 8 M urea and 50 mM Tris-HCl (pH 8.0) containing 5 mM DTT and incubated at 37 °C for 1 h. Alkylation was performed by adding iodoacetamide to a final concentration of 15 mM, followed by incubation in the dark at room temperature for 30 min. Samples were diluted 4-fold with 50 mM Tris-HCl (pH 8.0) to reduce the urea concentration to 2 M and digested overnight at 37 °C with trypsin (Promega) at an enzyme-to-protein ratio of 1:50 (w/w). Tryptic peptides were desalted using C18 ZipTip pipette tips (MilliporeSigma) according to the manufacturer's instructions. Briefly, ZipTips were prewetted with 50% acetonitrile, equilibrated with 0.1% formic acid, and peptides were bound to the resin. After washing with 0.1% formic acid, peptides were eluted with 80% acetonitrile in 0.1% formic acid. Eluted peptides were dried using a Vacufuge Plus vacuum concentrator (Eppendorf) operated in V-AL mode for 45 min at 30 °C and immediately analyzed.

### Absolute quantification of lysine acetylation by AQUA-MS

Absolute quantification (AQUA) of K153 acetylation in *Hv*GK was performed under different carbon source conditions using a targeted MS approach. The Ni-NTA-purified *Hv*GK samples were subjected to tryptic digestion, and synthetic heavy-isotope–labeled peptides representing the acetylated (AEWLLDNSDPIK(ac)LQR) and nonacetylated (AEWLLDNSDPIK) forms of the K153-containing tryptic peptide were used as external standards (Thermo Scientific). Parallel reaction monitoring analysis was performed on an Orbitrap Exploris 240 mass spectrometer coupled to a Thermo Vanquish Neo UHPLC system. Peptide separation was achieved using a 60-min LC gradient at a flow rate of 300 nl/min: 2 to 4% solvent B (0.1% formic acid in 80% acetonitrile) in 0.5 min, 4 to 28% B over 40 min, 28 to 42% B over 8 min, and 42 to 55% B in 4 min, followed by a final wash with 99% B for 7 min. The Orbitrap was operated at resolving powers of 120,000 (MS1) and 30,000 (MS2) at *m/z* 200 to monitor both the +2 charged light (m/z of 700.8564 and 920.4836) and heavy peptides (m/z of 704.8635 and 925.4877) mentioned above. Quantitative data analysis was performed using Skyline (MacCoss Lab Software) (51). Chromatographic peak areas of both endogenous and heavy peptides were extracted, and quantification was based on a standard curve generated from heavy peptide dilutions (200, 1000, 2000, 10,000, and 20,000 pg). Background signal was subtracted, and all peptide abundances were normalized to their respective spiked heavy peptides (2000 pg each). Linear regression (*R*^*2*^ > 0.99) confirmed high-quality quantification. The MS2 spectrum used for quantification corresponded to the y_8_ ion at m/z 920.48 for the acetylated peptide (precursor m/z 998.56) and m/z 700.85 for the nonacetylated form (precursor m/z 901.46). Acetylation occupancy was calculated as the ratio of the endogenous acetylated peptide to the total of acetylated and nonacetylated peptides.

### GK activity assay

*Hv*GK (ATP: glycerol 3-phosphotransferase, EC 2.7.1.30) enzymatic activity was measured in a coupled photometric assay, as previously described (3). *Hv*GK activity was coupled to the formation of α-glycerophosphate, which was quantified in the presence of NAD^+^ and commercially available α-glycerophosphate dehydrogenase. The primary reaction mixture (1 ml) contained purified *Hv*GK (0.84 μg), 3.5 mM MgCl_2_, 3.5 mM ATP, 4.6 mM glycerol, 50 mM HEPES (pH 8.0), and 0.1 M NaCl. Reaction linearity was confirmed by stopping replicate reactions at 20, 30, and 40 min, showing proportional glycerol-3-phosphate (G3P) formation and highly similar specific activities when normalized by time (R^2^ ≥ 0.99). Based on this, a 30-min incubation was selected as the midpoint of the linear range for kinetic analyses. Based on this, a 30-min incubation was selected as the midpoint of the linear range for kinetic analyses. Routine endpoint assays were incubated for 30 min at 57 °C and terminated by adding an equal volume of 0.2 N phosphoric acid, followed by centrifugation (10 min, 12,000×*g*). G3P levels were determined enzymatically as previously described (52). An aliquot of the terminated reaction (110 μl) was mixed in a total reaction volume of 1 ml containing 0.011 N NaOH for neutralization, 1.1 mM NAD^+^, 0.66 M hydrazine sulfate adjusted to pH 9.4 with NaOH, 1% (w/v) nicotinamide-sodium carbonate buffer, and 8 U of rabbit muscle α-glycerophosphate dehydrogenase (EC 1.1.1.8; Sigma, 500 U). After 1 h incubation at 30 °C, NADH production was measured at 340 nm. G3P concentrations were calculated by linear regression analysis (R^2^ > 0.99) using standards linear between 0 and 5 mM G3P.

### Size-exclusion chromatography

Purified *Hv*GK samples were dialyzed into 50 mM HEPES (pH 7.5), 2 M NaCl, 1 mM DTT, and 10% glycerol. Proteins (100 μl) were loaded onto Superdex 200 HR 10/300 Gl columns at 0.3 ml/min. Molecular masses were estimated using standard curves generated from known molecular weight standards: Thyroglobulin (bovine) (670 kDa), γ-globulin (bovine) (158 kDa), ovalbumin (chicken) (44 kDa), myoglobin (horse) (17 kDa), and vitamin B12 (1.35 kDa) (Bio-Rad Laboratories).

### Differential scanning fluorimetry thermal shift assay

Thermal shift assays were performed to assess the impact of ligand binding on the stability of His-*Hv*GK K153Q and K153R variants compared to wt. The proteins were purified from *H*. *volcanii* KM03 strains grown in GlyMM. Differential scanning fluorimetry experiments were conducted as previously described (3). Briefly, each reaction contained 3 μM monomeric enzyme in a final volume of 25 μl, prepared in buffer supplemented with various combinations of 35 mM MgCl_2_, 46 mM glycerol, and 35 mM ATP. SYPRO Orange protein gel stain (Invitrogen, Cat. No. S6650) was added to a final concentration of 2.5 × . Enzyme-minus controls were used to correct for background fluorescence. Thermal unfolding was monitored using a C1000 Touch Thermal Cycler coupled to a CFX96 Real-Time PCR Detection System (Bio-Rad Laboratories). Samples were subjected to a linear temperature gradient from 20 °C to 95 °C at a rate of 1 °C per min. Fluorescence was recorded continuously, and T_m_ were determined by identifying the peak of the first derivative of the fluorescence intensity with respect to temperature. Comparisons across different ligand conditions were made to evaluate their effects on the thermal stability of the His-*Hv*GK K153Q and K153R variant proteins.

### Kinetic analysis of H*v*GK variants

Kinetic assays were conducted using purified His-*Hv*GK variants: wt, K153Q, and K153R, expressed in the *H*. *volcanii* KM03 strain (*ΔglpK* *ΔlarC*). Reactions were performed under optimized conditions: 100 mM NaCl, pH 8.0 (HEPES buffer), and 57 °C. *Hv*GK activity was measured using the coupled enzymatic assay that monitored NADH oxidation at 340 nm. Substrate concentrations were varied to assess their effect on enzyme kinetics: glycerol (0, 0.2, 0.4, 0.6, 0.8, 1, 2, 3, and 4.6 mM) and ATP (0, 0.2, 0.4, 0.6, 0.8, 1, 2, 3, 3.5, 4, 4.5, 5, 5.5, 6, 6.5, and 7 mM). Kinetic parameters (*K*_m_, *K*_*D*_, *V*_max_, *k*_cat_, *k*_cat_*/K*_m_, and Hill coefficient *n*) were calculated by nonlinear regression fitting to the Michaelis–Menten, Lineweaver–Burk, and Hill equations using Excel Solver for iterative optimization. For the K153R variant with ATP, both sigmoidal and Michaelis–Menten models were tested to determine the best fit. All assays were performed in three experimental replicates, and results are presented as mean ± standard deviation.

### Growth plate assay to assess the impact of H*v*GK lysine acetylation on carbon source utilization

To evaluate the impact of *Hv*GK lysine acetylation on carbon source utilization, KM06 (H1207 *ΔglpK* *ΔlarC* *Δpat2*) was used as a host. KM06 strains were generated through transformation to individually carry the following plasmids: pJAM202c (empty vector), pJAM4351 (His-*Hv*GK), pJAM4354 (His-*Hv*GK K153Q acetylation mimic), and pJAM4355 (His-*Hv*GK K153R nonacetylated variant). The strains were cultivated in ATCC 974 supplemented with novobiocin to stationary phase and stored in 20% (w/v) glycerol stocks at −80 °C. For assay, strains were inoculated from the freezer stocks using a loop onto GlyMM or GluMM plates. Plates were incubated at 42 °C for 8 days. Growth was monitored by streak/colony formation. Growth on GlyMM was used as an indicator of functional complementation by the respective His-*Hv*GK variants in the absence of the *pat2* gene.

### Analysis of H*v*GK abundance and acetylation in acetyltransferase and deacetylase mutant strains

To assess the role of different acetyltransferases or deacetylases in the abundance and acetylation state of *Hv*GK, *H*. *volcanii* strains lacking acetyltransferase or deacetylase genes were generated and analyzed. Strains KM05 (*Δpat1*), KM06 (*Δpat2*), KM07 (*Δsir2*), and KM08 (*Δelp3*) were each transformed with plasmids expressing His-*Hv*GK or the empty vector pJAM202c as a control. Cultures were grown in ATCC, GluMM, FruMM, and GlyMM media at 42 °C with orbital shaking (200 rpm) to stationary phase (OD_600_ > 1.0). Aliquots corresponding to 0.5 OD_600_ units were harvested by centrifugation, and cell pellets were stored at −80 °C until further analysis. For protein analysis, pellets were resuspended directly in 2 × Laemmli sample buffer, boiled for 5 min, and centrifuged at RT (10 min, 13,000×*g*). The equivalent of 0.1 *A* of each sample was loaded onto the SDS-PAGE gels. Proteins were resolved by SDS-PAGE and analyzed by CB staining, as well as IB using anti-His and anti-acetyllysine (Kac) antibodies to assess His-*Hv*GK abundance and acetylation status. This experimental setup enabled direct evaluation of the impact of acetyltransferase and deacetylase deletions on His-*Hv*GK expression and acetylation *in vivo*.

### Statistical analysis

All experiments included technical replicates performed in at least triplicate and were independently repeated at least three times to ensure reproducibility. Data are presented as mean values ± standard deviation. Statistical significance between experimental groups was determined using Student's *t* test, with *p* < 0.05 considered statistically significant. All statistical analyses were performed using Microsoft Excel.

## Data availability

The MS-based proteomic datasets generated in this study are publicly available in the UCSD MassIVE repository (Mass Spectrometry Interactive Virtual Environment, https://massive.ucsd.edu) under the following accession numbers ID: MSV000097918, MSV000097919, MSV000097920, and MSV000097921. Each dataset includes the raw MS files used for peptide identification and quantification.

## Supporting information

This article contains supporting information with citations: (2, 3, 9, 27, 45, 46, 53, 54, 55).

## Conflict of interest

The authors declare that they have no conflicts of interest with the contents of this article.

## Figures and Tables

**Figure S2:**
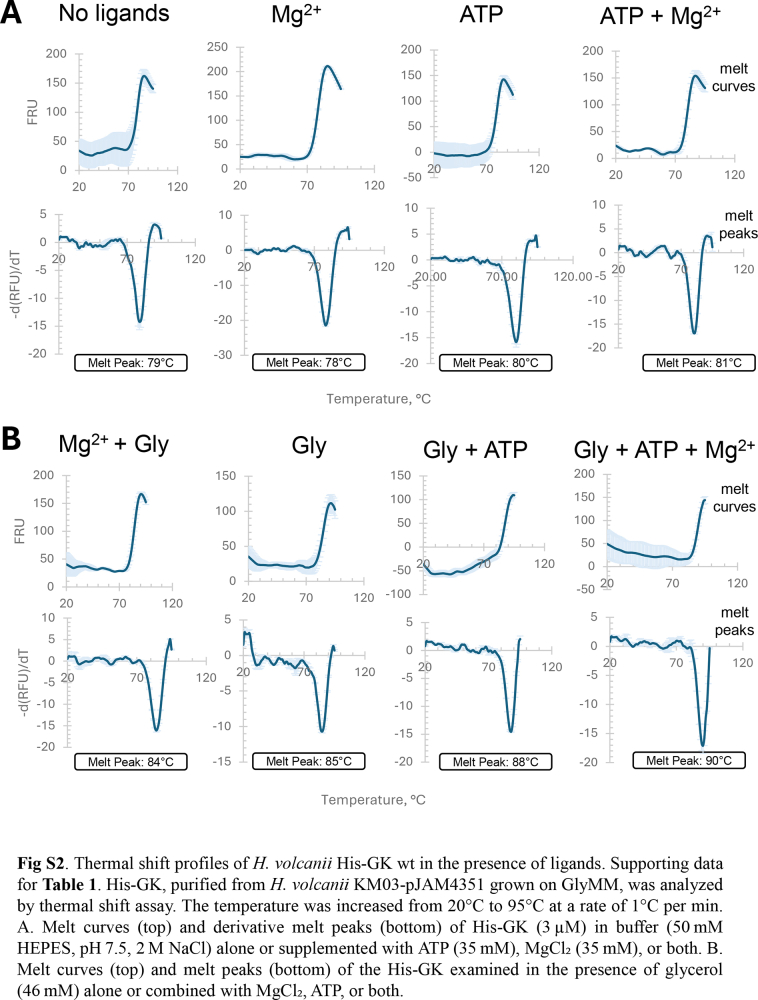


**Figure S3:**
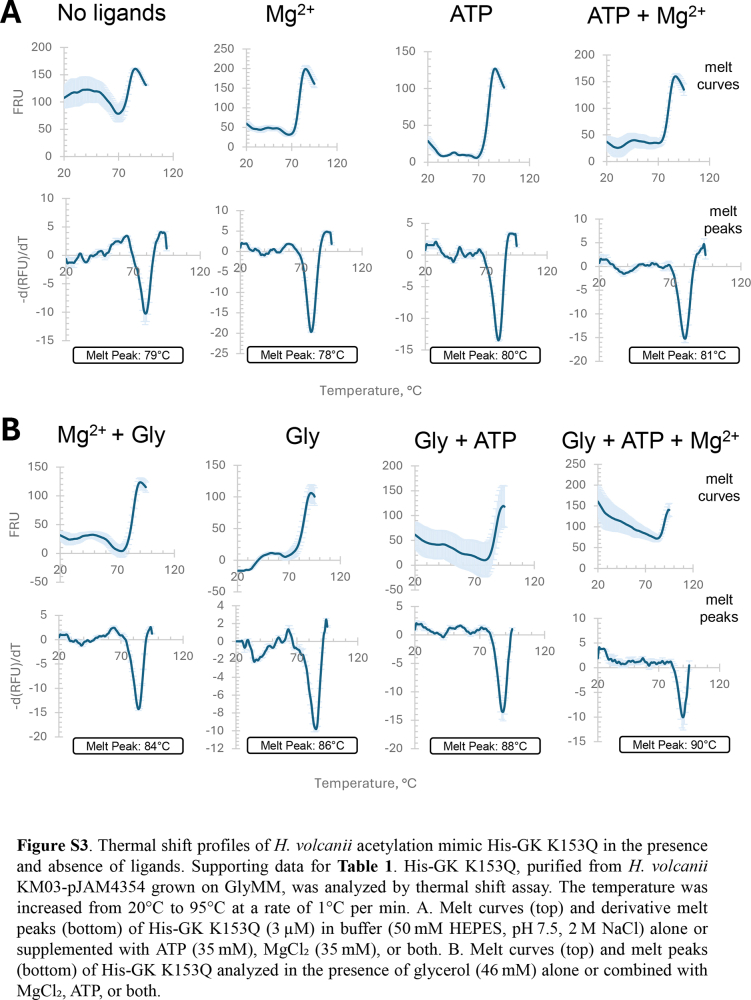


**Figure S4:**