# Supplementary material for: Dynamic acetylation of a conserved lysine impacts glycerol kinase activity and abundance in the haloarchaeon Haloferax volcanii
Source: J Biol Chem. 2025 Nov 20;302(1):110960. doi: 10.1016/j.jbc.2025.110960 (PMC12853182; doi:10.1016/j.jbc.2025.110960)
Supplement: Table S1 [file mmc1.pdf]

Table S1. List of strains and plasmids used in this study

Strain or plasmid	Description	Source or Ref.
***E. coli* strains**		
Top 10	F– *recA1 endA1 hsdR17*(r_K–m_K+) *supE44 thi-1 gyrA relA1*	Invitrogen
GM2163	F– *ara-14 leuB6 fhuA31 lacY1 tsx78 glnV44 galK2 galT22 mcrA dcm-6 hisG4 rfbD1rpsL136 dam13::*Tn*9 xylA5 mtl-1 thi-1 mcrB1 hsdR2*	New England Biolabs
Rosetta (DE3)	F– *ompT hsdSB*(rB–mB–) *gal dcm lacY1*(DE3) pRARE (Cam^r)	Novagen/ MilliporeSigma
***H. volcanii* strains**		
H26	DS70 Δ*pyrE2*	(45)
H1207	Δ*pyrE2* $pitA_{Nph}$ Δ*mrr*	(53)
KS4	H26 Δ*glpK*	(2)
KM01	H1207 Δ*glpK*	(3)
KM03	H1207 Δ*glpK* Δ*larC*	This study
KM05	H1207 Δ*glpK* Δ*larC* Δ*pat1*	This study
KM06	H1207 Δ*glpK* Δ*larC* Δ*pat2*	This study
KM07	H1207 Δ*glpK* Δ*larC* Δ*sir2*	This study
KM08	H1207 Δ*glpK* Δ*larC* Δ*elp3*	This study
**Plasmids**		
pTA131	Ap^r; pBluescript II containing P*fdx-pyrE2*	(45)
pJAM202c	Ap^r Nv^r; empty vector derived from plasmid pBAP5010	(54)
pJAM503	Ap^r Nv^r; pBAP5010 containing $P2_{rrn}$-*his6-panA*	(54)
pET15b	Ap^r; *E. coli* expression plasmid vector	Novagen
pJAM4351	$Ap^r Nv^r$; pJAM503-derived expression plasmid containing $P2_{rrn}$-*his6-glpK*	This study
pJAM4354	$Ap^r Nv^r$; pJAM503-derived expression plasmid containing $P2_{rrn}$-*his6-glpK* K153Q	This study
pJAM4355	$Ap^r Nv^r$; pJAM503-derived expression plasmid containing $P2_{rrn}$-*his6-glpK* K153R	This study
pJAM4360	Ap^r; pET15b-derived expression plasmid containing *his6-glpK*	This study
pJAM4361	Ap^r; pET15b-derived expression plasmid containing *his6-glpK* K153Q	This study
pJAM4362	Ap^r; pET15b-derived expression plasmid containing *his6-glpK* K153R	This study
pJAM4357	Apr; pTA131 carries *larC* and 500 bp flanking sequences (pre-knockout plasmid)	This study
pJAM4358	Ap^r; pJAM4357 *ΔlarC* (knockout plasmid)	This study
pJAM4013	Ap^r; pTA131 carrying *pat1* 500 bp flanking sequence. *Δpat1* knockout plasmid	(9)
pJAM4014	Ap^r; pTA131 carrying *pat2* 500 bp flanking sequence. *Δpat2* knockout plasmid	(9)
pJAM4015	Ap^r; pTA131 carrying *pat1* 500 bp flanking sequence. *Δsir2* knockout plasmid	(9)

pJAM4464	Ap^r; pTA131 carrying *pat1* 500 bp flanking sequence. *Δelp3* knockout plasmid	(55)

Ap^r, ampicillin resistance; Nv^r, novobiocin resistance; $Cam^{r,}$ chloramphenicol resistance; *his6*-, encodes N-terminal His-tag; *glpK*, encodes glycerol kinase (GK, HVO_1541); Δ*sir2*, Δ*hvo_2194*; Δ*pat1*, Δ*hvo_1756*; Δ*pat2*, Δ*hvo_1821*; *Δelp3, hvo_2888*.
