# Supplementary material for: Dynamic acetylation of a conserved lysine impacts glycerol kinase activity and abundance in the haloarchaeon Haloferax volcanii
Source: J Biol Chem. 2025 Nov 20;302(1):110960. doi: 10.1016/j.jbc.2025.110960 (PMC12853182; doi:10.1016/j.jbc.2025.110960)
Supplement: Table S2 [file mmc2.pdf]

Table S2. List of primers used in this study

Primer	Sequences (5' - 3')	Description	Ref.
F-glpK	GGTCATATGTCAGGAGAAACTTACGTCGG	HVO_1541 pJAM503 and pET15b; NdeI	(27)
R- glpK	TTTGCTCAGCTTATTCCTCCCGTGCCC	HVO_1541 pJAM503 and pET15b; BlpI	(27)
F-*glpK*	CAGGCACGCGGTTCATGG	HVO_1541 pJAM503	This study
R-*glpK*_K153R	CGCTGGAGCcTGATGGGG	HVO_1541 pJAM503 SDM AAG-AGG	This study
R-*glpK*_K153Q	GCTGGAGCtgGATGGGGTC	HVO_1541 pJAM503 SDM AAG-CAG	This study
F- HvPrrn	CGATGCCCTTAAGTACAACAGGGT	pJAM503 specific primer	(46)
R- T7Ter	AACCCCTCAAGACCCGTTTAGAG	pJAM503 specific primer	(46)
F-NphpitA	GAATTCATGCCACAACGCCAACCAC	$pitA_{Nph}$ forward primer; EcoRI	(53)
R-NphpitA	TCTAGATCAGGCGAGGAAGACGTGG	$pitA_{Nph}$ reverse primer; XbaI	(53)
F-pitA	GGAAAATCAAGCAGGTCATCGC	Forward primer specific to $pitA_{Hvo}$	(53)
R-pitA	GTAGAACATCCCCATCGTGCC	Reverse primer specific to $pitA_{Hvo}$	(53)
F-NphPitA	GCAGTATGCCGACAAGGTCTCC	Forward primer specific to $pitA_{Nph}$	(53)
R-NphPitA	CCCGCTCGTTTTTCCACAG	Reverse primer specific to $pitA_{Nph}$	(53)
F- pTA131	CGTCAAAGGGCGAAAAACCGTC	pTA131 specific primer	This study
R- pTA131	CACTCATTAGGCACCCCAGGCTT	pTA131 specific primer	This study
F- larC	CGAGGATCCAGCACGAGTTTGTCGGCCTTCGTAG	HVO_2381 pTA131; BamHI	This study
R- larC	TTAAAGCTTGTTCGCATCGGCGACGGCGCG	HVO_2381 pTA131; HindIII	This study
F-rev larC	CTATGAACCCGACGCGGCGCG	Reverse PCR; HVO_2381 deletion	This study
R-rev larC	GCGTAAACCGTGGTGTTCGAGCGTA	Reverse PCR; HVO_2381 deletion	This study
F-ver larC	GACATCAGTGTCCTCGGTGACGAGG	HVO_2381 deletion verification	This study
R-ver larC	CACGGGCTCCGACGGGAACGT	HVO_2381 deletion verification	This study

F- Pat1	ATCGGATCCGCGTTGCCGAGGTAGAAGAACGTC	HVO_1756 pTA131 KO; BamHI for deletion verification	(9)
R- Pat1	TTTAAGCTTCGAACGCGGACTGAGCGCCTCGGA	HVO_1756 pTA131 KO; HindIII for deletion verification	(9)
F- Pat2	TTTGGATCCGGACTCGTCTGTCATACCGCGGGC	HVO_1821pTA131 KO; BamHI for deletion verification	(9)
R- Pat2	TTTAAGCTTCGCGCCCGCTCTCTATCGACCTCG	HVO_1821 pTA131 KO; HindIII for deletion verification	(9)
F- Sir2	TTCGGATCCCCTCGTCGGGCCACTCGTCC	HVO_2194 pTA131 KO; BamHI for deletion verification	(9)
R- Sir2	TTAGGTACCGACCGCCGCCCGAACCCGG	HVO_2194 pTA131 KO; KpnI for deletion verification	(55)
F- Elp3	CGCGGATCCCGCGTACGAGTCCAGTTTCT	HVO_2888 pTA131 KO; BamHI for deletion verification	(55)
R- Elp3	CCGCTCGAGGTCGCGTTGGAAGCCTACTA	HVO_2888 pTA131 KO; XhoI for deletion verification	(55)

Restriction endonuclease sites used in cloning are underlined. F, forward. R, reverse; Primer sequence, lowercase letters indicate modified bases designed for site-directed mutagenesis; SDM, site-directed mutagenesis; KO, knockout.
