# Supplementary material for: Dynamic acetylation of a conserved lysine impacts glycerol kinase activity and abundance in the haloarchaeon Haloferax volcanii
Source: J Biol Chem. 2025 Nov 20;302(1):110960. doi: 10.1016/j.jbc.2025.110960 (PMC12853182; doi:10.1016/j.jbc.2025.110960)
Supplement: Dataset S1 [file mmc3.docx]

**Dataset S1.** FASTA files of the amino acid sequences of glycerol kinase (GK) homologs used for multiple sequence alignment.

>sp|P32189|GLPK_HUMAN Glycerol kinase OS=Homo sapiens OX=9606 GN=GK PE=1 SV=3

MAASKKAVLGPLVGAVDQGTSSTRFLVFNSKTAELLSHHQVEIKQEFPREGWVEQDPKEI

LHSVYECIEKTCEKLGQLNIDISNIKAIGVSNQRETTVVWDKITGEPLYNAVVWLDLRTQ

STVESLSKRIPGNNNFVKSKTGLPLSTYFSAVKLRWLLDNVRKVQKAVEEKRALFGTIDS

WLIWSLTGGVNGGVHCTDVTNASRTMLFNIHSLEWDKQLCEFFGIPMEILPNVRSSSEIY

GLMKISHSVKAGALEGVPISGCLGDQSAALVGQMCFQIGQAKNTYGTGCFLLCNTGHKCV

FSDHGLLTTVAYKLGRDKPVYYALEGSVAIAGAVIRWLRDNLGIIKTSEEIEKLAKEVGT

SYGCYFVPAFSGLYAPYWEPSARGIICGLTQFTNKCHIAFAALEAVCFQTREILDAMNRD

CGIPLSHLQVDGGMTSNKILMQLQADILYIPVVKPSMPETTALGAAMAAGAAEGVGVWSL

EPEDLSAVTMERFEPQINAEESEIRYSTWKKAVMKSMGWVTTQSPESGDPSIFCSLPLGF

FIVSSMVMLIGARYISGIP

>sp|P0A6F3|GLPK_ECOLI Glycerol kinase OS=Escherichia coli (strain K12) OX=83333 GN=glpK PE=1 SV=2

MTEKKYIVALDQGTTSSRAVVMDHDANIISVSQREFEQIYPKPGWVEHDPMEIWATQSST

LVEVLAKADISSDQIAAIGITNQRETTIVWEKETGKPIYNAIVWQCRRTAEICEHLKRDG

LEDYIRSNTGLVIDPYFSGTKVKWILDHVEGSRERARRGELLFGTVDTWLIWKMTQGRVH

VTDYTNASRTMLFNIHTLDWDDKMLEVLDIPREMLPEVRRSSEVYGQTNIGGKGGTRIPI

SGIAGDQQAALFGQLCVKEGMAKNTYGTGCFMLMNTGEKAVKSENGLLTTIACGPTGEVN

YALEGAVFMAGASIQWLRDEMKLINDAYDSEYFATKVQNTNGVYVVPAFTGLGAPYWDPY

ARGAIFGLTRGVNANHIIRATLESIAYQTRDVLEAMQADSGIRLHALRVDGGAVANNFLM

QFQSDILGTRVERPEVREVTALGAAYLAGLAVGFWQNLDELQEKAVIEREFRPGIETTER

NYRYAGWKKAVKRAMAWEEHDE

>sp|P18157|GLPK_BACSU Glycerol kinase OS=Bacillus subtilis (strain 168) OX=224308 GN=glpK PE=1 SV=2

METYILSLDQGTTSSRAILFNKEGKIVHSAQKEFTQYFPHPGWVEHNANEIWGSVLAVIA

SVISESGISASQIAGIGITNQRETTVVWDKDTGSPVYNAIVWQSRQTSGICEELREKGYN

DKFREKTGLLIDPYFSGTKVKWILDNVEGAREKAEKGELLFGTIDTWLIWKMSGGKAHVT

DYSNASRTLMFNIYDLKWDDELLDILGVPKSMLPEVKPSSHVYAETVDYHFFGKNIPIAG

AAGDQQSALFGQACFEEGMGKNTYGTGCFMLMNTGEKAIKSEHGLLTTIAWGIDGKVNYA

LEGSIFVAGSAIQWLRDGLRMFQDSSLSESYAEKVDSTDGVYVVPAFVGLGTPYWDSDVR

GSVFGLTRGTTKEHFIRATLESLAYQTKDVLDAMEADSNISLKTLRVDGGAVKNNFLMQF

QGDLLNVPVERPEINETTALGAAYLAGIAVGFWKDRSEIANQWNLDKRFEPELEEEKRNE

LYKGWQKAVKAAMAFK

>tr|A0AAX3AIS8|A0AAX3AIS8_HALDO Glycerol kinase OS=Halococcus dombrowskii OX=179637 GN=glpK PE=3 SV=1

MSEGTYVGAIDQGTTGTRFMVFDHGGGVVASAYEKHEQYYPEPGWVEHDPMEIWENTKSV

IADALDSAGIDATRLDAIGVTNQRETTLFWDADTHRPIMRAIVWQDRRTTDRIEELETNG

KAETIQAKTGLEPDAYFSATKAEWILDNADPIKLQRSRPQDIRERAADGEVRFGTIDSWL

IENLTGNHITDVTNASRTMLFNIHEMDWDDELLEEFRVPRAALPEVRPSSDENLYGTTDA

DGFLGAEIPVAGALGDQQAALFGQTCFDPGDAKNTYGTGSFFLLNTGEEAVESDHGLLTT

IGFQRSGEPVQYALEGSIFVTGAAIEWLEDVELLDNAMESETVARSVESTDGVYLVPAFT

GLGAPHWDGRARGTIVGMTRGTERAHIVRAALESIAFQTRDVAEAMEADAGIDISSLQVD

GGAVKNNFLCQLQANIVGTDIVRPEVDETTALGSAYAAGLAVGYWDDPEKLRDNWRVDRE

FTPDEDASVDGRYERWSEAVERSRDWAREA

>tr|A0AAV3UDB2|A0AAV3UDB2_9EURY Glycerol kinase OS=Haladaptatus pallidirubidus OX=1008152 GN=glpK PE=3 SV=1

MKQNETYVGSIDQGTTGTRFMVFDHGGAVVANAYEKHEQIYPEPGWVEHDPTEIWENTKS

VIQSALAEAGIQADQLAAIGVTNQRETTLLWDADTGKPIHNAIVWQDRRTTNRIERLEKD

GRTDDVRAKTGLEPDAYFSATKAEWLLDNSEPIKTQRARPADLRDRAETGEILFGTIDSW

LIYNLTGEHITDVTNASRTMLFDIHEMEWDDELCEEFRVPREMLPEVRPSSDDDTYGSTD

SDGFLGAEVPVAGALGDQQAALFGQTCFDAGDAKNTYGTGSFFLLNTGEEAVTSEHGLLT

TVGFQRSGEPVQYALEGAIFVTGAAIEWLVDMDLIDDAIETENLARSVDSTDGVFMVPAF

TGLGAPHWNQRARGTLVGMTRGTRKEHVVRATLESIAFQTRDVAEAMEADSGIEVESLRV

DGGAVKNNFLCQLQADILGTDIVRPVVDETTALGSAYAAGLAVDYWETVDELRNNWQVDR

EFDAGTELGTADVDEKYGRWQDAVSRSLDWAQEGSD

>tr|E4NLP8|E4NLP8_HALBP Glycerol kinase OS=Halogeometricum borinquense (strain ATCC 700274 / DSM 11551 / JCM 10706 / KCTC 4070 / PR3) OX=469382 GN=glpK PE=3 SV=1

MTENTYIGAVDQGTTGTRFMVFDHSGKVVASAYQKHEQIYPEPGWVEHAPAEIWENTQAV

MLTALEEAGLDAEQLEAIGITNQRETTLIWDKASGRAIANAIVWQDRRTTDRVETLQDEG

KEEWVREKTGLEPDAYFSATKAEWLLDNADPIKLARMRPDDVRERAEQGELMFGTIDSWV

TYKLTGAHVTDVTNASRTMLFNIHDMDWDDELLEEFNVPAELLPEVRPSSDEDLYGTTDP

DGFLGAEIPVAGALGDQQAALFGQTCFDAGDAKNTYGTGSFMLMNTGDEAVMSEHGLLTT

VGFQRSGEPVQYALEGSIFITGAAIEWLEDVSLIDDAAETEELARSVDSTDGVYFVPAFT

GLGAPHWDQRARGTILGMTRGTRREHVVRATLESIAFQTRDVAEAMEEDSGIDLSTLRVD

GGAVKNNFLCQLQSNIVGTDIVRPMVDETTALGAAYAAGLAVGYWSNLDELRDNWQIDRE

FSPDDSAENVDGRYGRWKEAVDRSRDWAREGSD

>tr|A0A1H8DYP7|A0A1H8DYP7_9EURY Glycerol kinase OS=Halorientalis persicus OX=1367881 GN=glpK PE=3 SV=1

MTANTYVGAIDQGTTGTRFMVFDHGGQVVANAYEKHEQVYPEPGWVEHDAVEIWENVKTV

ITRALEEAGLDATQLAALGITNQRETTVVWDAETGDPIHNAIVWQDRRTTDRVERLEAEG

KLEWIREKTGLEADAYFSATKAEWLLDNADPIKMERTRTEDVRDRAEAGELLLGTIDAWL

IYNLTGNHVTDVTNASWTMLYDVHDMAWCADLLAEFSVPRAMLPEVRPSSDENHYGYTDP

DGFLGTSVPVAGALGDQQAALFGQTCFDAGDAKNTYGTGSFFLMNTGEQAVESDHGLLTT

VGFQRSGGPVQYALEGSIFVTGAAIEWLEDVDLISSPAETAELARSVDGTDGVYVVPAFT

GLGAPHWDGRARGTIVGMTRGTRREHIVRATLEAIAYQTRDVAEAMEADAGVEMGSLRVD

GGAVKNNFLCQLQSDIIGTEIVRPEVDETTALGAAYAAGLAVGYWDSLAELRNNWHVDAE

FAPEMDRSDADARFERWHDAVERSKDWAQEGGD

>sp|B0R6S2|GLPK_HALS3 Glycerol kinase OS=Halobacterium salinarum (strain ATCC 29341 / DSM 671 / R1) OX=478009 GN=glpK PE=3 SV=1

MTDAYVGAIDQGTTGTRFIVFDQHGDVVANTYEKHEQHYPEPGWVEHDPLEIWENTKSVV

TAGLSAAGLDADDLAAIGITNQRETTVVWDAASGRPIHNALVWQDRRTTSRVESLEENGK

IERIREKTGLEADAYFSATKTEWLLDEAEPLKLSSARASSLRDRARDGELLMGTIDSWLI

YNLTGEHITDVSNASRTMLYNITDLEWDDWLLEEFDIPREMLPEVRPSSDEAVYGHTDPD

GFLGAAVPVTAALGDQQAALFGQTCFDAGDAKNTYGTGSFYLMNTGEDAVSSEHGLLTTI

GFQLSGEPVQYALEGSIFVTGAAIEWLEDVDLINNAAQTAELASSVDTTDGVYMVPAFTG

LGAPHWDGRARGTLVGMTRGTRKAHIVRATLESIAYQTRDIAAAMEADSGVSTTTLRVDG

GAVKNNFLCQLQSDIIQTDLARPEVDETTALGAAYAAGLAVGYWDSLDDLRENWRVDRSF

EPEMDPSEADSKYGRWEDAVDRSLAWATED

>tr|A0A897MXY4|A0A897MXY4_9EURY Glycerol kinase OS=Halapricum desulfuricans OX=2841257 GN=glpK PE=3 SV=1

MSDTYVGAIDQGTTGTRFMVFDHDGTVAASAYEKHEQIYPEPGWVEHDPMEIWENTKDVV

IEALAQAGIEASQLEAIGVTNQRETTIVWDAETGKPVHNAIVWQDRRTTDRVEELQEEDK

VEWIREKTGLEADAYFSATKVEWLLEEGDPVKMQRSRPQDNKERAEAGELMMGTPDAWII

YNLTGNNITDVTNASRTMLYNIRDMEWDEELLEEFGVPEQMLPEVRPSSDEDYYGHTDAD

GFLDAEVPVAGALGDQQAALFGQTCFDAGDAKNTYGTGSFFLMNTGEEAVKSDHGLLTTV

GFQRSGEPVQYALEGSIFITGAAIEWLEDVELIDDPTETAELARSVDSTDGVYMVPAFTG

LGAPHWDGRARGTIVGMTRGTRREHIVRATLESIAYQTRDVAEAMEDDSGVELTSLKVDG

GAVKNNFLVQLQSDIIQTDIARPQVDETTALGAAYAAGLAVGYWETLDELRDNWQVDREF

EPDMDPAKADKLHDRWEDAVERSLDWATEE

>tr|A0A7J9SFV9|A0A7J9SFV9_9EURY Glycerol kinase OS=Halobellus ruber OX=2761102 GN=glpK PE=3 SV=1

MRDTYVGSIDQGTTGTRFIAFDHAGRVVASAYEKHEQIYPEPGWVEHDPIEIWENTKAVI

TRGLDDAGIDADQLAALGITNQRETTVVWDRETGRPVHNALVWQDRRTTDRVEELTEAGK

AEEIRAKTGLEVDAYFSATKTEWLLDNAEPLKMRSARGDDLRERAEAGELLMGTIDTWLV

YNLTGNHVTDVTNAARTMLFDIHDIAWNDDLLAEFDVPAAMLPEVRPSSDEELYGHTDPD

GFLGAEVPVAAALGDQQAALFGQTCFDEGDAKNTYGTGSFYLMNTGSEAVESDHGLLTTI

GFQLSGEPVQYALEGSIFITGAAIEWLEDVDLINNAAQTAELARSVDSTDGVYLVPAFTG

LGAPHWDGRARGTIVGMTRGTRKEHIVRATLESIAYQTRDVAEAMEADSGITTTSLRVDG

GAVKNDFLCQLQADILGLDIARPEVDETTALGSAYAAGLAVGYWDTLDELRDNWEVDRAF

AREMSGAVADERYGRWGEAVERSLDWAGEE

>tr|A0A1I6LTC7|A0A1I6LTC7_9EURY Glycerol kinase OS=Halomicrobium zhouii OX=767519 GN=glpK PE=3 SV=1

MTDTYVGAIDQGTTGTRFMVFDHGGQVVANAYEKHEQIYPEPGWVEHDPVEIWENTKSVV

GRALDEADVEADQLAAIGITNQRETTLVWDHDSGDPVHNALVWQDRRTTDRVEELEAEDK

VEWIRGKTGLEADAYFSATKVEWILDNADPLKLEASRPENLRDRAEAGELRMGTIDTWLI

SKLTGNVITDVTNASRTMLYDIHDMDWDDELLEEFGVPWSMLAEVRPSSDEDLYGHTDPD

GFLGAEIPVAGALGDQQAALFGQTCFDEGDAKNTYGTGSFFLMNTGNEAVDSEHGLLTTV

GFQRSGEPVQYALEGSIFITGAAIEWLEDVDLITNAAQTAELASSVDSTDGVYMVPAFTG

LGAPHWDGRARGTIVGMTRGTRKEHIVRATLESIAYQTRDVAEAMEADSGIEMGQLRVDG

GAVKNNFLCQLQADIIRTGIVRPEVDETTALGSAYAAGLAVGYWESVDELRDNWHVDREF

TPEMDAARADRLYDRWGDAVERSLDWAREE

>tr|C7NNX3|C7NNX3_HALUD Glycerol kinase OS=Halorhabdus utahensis (strain DSM 12940 / JCM 11049 / AX-2) OX=519442 GN=glpK PE=3 SV=1

MTDTYVGAIDQGTTGTRFMVFDRDGQVAGNAYEKHEQFYPEPGWVEHDPLEIWANTKAVV

TAGLADAGLEAEQVAALGITNQRETTLVWDKDTGKPVHNALVWQDRRTTDRVEELQDEDK

VEWIRGKTGLEPDAYFSATKTEWILDNAEPLKLQSHRASDLHDRAESGELLMGTIDAWLI

YNLTGNHITDVTNASRTMLYNVHEMAWDGDLLAEFGVPEAMLPEVRPSSDENLYGHTDPD

GFLGAEIPVAGALGDQQAALFGQTCFEEGDAKNTYGTGSFYLMNTGTEAVESDHGLLTTI

AFQRSGEPVRYALEGSIFATGAAIEWLEDVDLINNAAQTADLASAVDSTDGVYLVPAFTG

LGAPHWDGRARGTIVGMTRGTRKEHIVRATLEAIAYQTRDVAEAMEADSGIETTTLRVDG

GAVKNDFLCQLQADVIRTDIARPEVDETTALGSAYAAGLAVGYWADLDELRENWRVDREF

TPEIPVEDADRMYSRWDDAVERARDWAREE

>sp|Q18JE8|GLPK_HALWD Glycerol kinase OS=Haloquadratum walsbyi (strain DSM 16790 / HBSQ001) OX=362976 GN=glpK PE=3 SV=1

MADTYVGSIDQGTTGTRFMVFDHSGQVVANAYEKHEQIYPNPGWVEHDPIEIWENTKEVV

TRGLEEAGLDAEQLEALGVTNQRETTIVWDEASGKPVHNALVWQDRRTTDRVEEIQEAGK

VEMIREKTGLECDAYFSATKTEWILDNAEPLKMQASRGGDVRDRAEDGELLMGTIDSWLI

QNLTGNHITDVTNASRTMLYNIRELEWDDELLEEFRVPRSMVPEVRPSSDDEYYGHTDAD

GFLGAEIPVAGALGDQQAAMFGQTCFDEGDAKNTYGTGSFYLMNTGTDAVASDHGLLTTI

GFQMSGEPVQYALEGSIFVTGAAIEFLEDVDLINNAAQTAELASSVDSTDGVYMVPAFTG

LGAPHWDGRARGTLVGMTRGTEKEHIVRATLESIGYQTRDVAEAMEADSGIETTSLRVDG

GAVKNNFLCQLQSDILQTDIVRPVVDETTALGSAYAAGLAVGYWDTVDELRDNWQVDREF

ESEMDSADANTMYDRWDDAVERSLDWAQEE

>tr|A0A7D5P6V2|A0A7D5P6V2_9EURY Glycerol kinase OS=Halosimplex rubrum OX=869889 GN=glpK PE=3 SV=1

MADTYIGAIDQGTTGTRFMVFDHEGQVVANAYEQHEQIYPQPGWVEHDPIEIWENTQEVV

TRGLRDGGLDATQLEALGITNQRETTVVWDAETGKPVHNAIVWQDRRTTDRVEQLEAEDK

VEWIREKTGLEADAYFAATKTEWILDNAEPLKLQSSRGESVRDRAEAGELRMGTIDTWLI

YNLTGEHITDVTNASRTMLYDIEGLSWDPELLEEFGVPESMLPEVRPSSDEATYGSTDPD

GFLGAEVPVAGALGDQQAALFGQTCFDAGDAKNTYGTGSFYLMNTGDEAVASEHGLLTTI

GFQLSGEPVQYALEGSIFITGAAIEWLEDVDLINNAAQTAELARSVESTDGVYMVPAFTG

LGAPHWDGRARGTIVGMTRGTRKEHIVRATLESIAYQTRDIAEAMEADSGVETTSLRVDG

GAVKNNFLCQLQSDIIQTEIVRPEVDETTALGSAYAAGLAVGYWDSVDELRDNWQVDREF

SPEMSGEEADRMYGRWDDAVERSLDWAREE

>sp|Q5V4I4|GLPK_HALMA Glycerol kinase OS=Haloarcula marismortui (strain ATCC 43049 / DSM 3752 / JCM 8966 / VKM B-1809) OX=272569 GN=glpK PE=3 SV=1

MADTYVGAIDQGTTGTRFMVFDHSGQVVANAYEKHEQIYPEPGWVEHDPVEIWENTQEVV

TKGLADAGVGAEQLEALGITNQRETTIVWDKETGKPVHNALVWQDRRTTDRVEEIQEEDK

VEWIRGKTGLECDAYFSATKTEWILDNAEPLKMQSSRGADLRERAEDGELLMGTIDAWLI

YKLTGNHITDVSNASRTMLYNIHDMEWDDELLEEFGVPESMVPEVRPSSDESLYGHTDAD

GFLKEEVPVAGALGDQQAALFGQTCFDKGDAKNTYGTGAFYLMNTGSEAVASDNGLLTTV

GFQMSGEPVQYALEGSIFIAGAAIEWLEDVDLINNAAQTAELARSVESTDGVYMVPAFTG

LGAPHWDGRARGTIVGMTRGTRKEHIVRATLESIAYQTRDLAEAMEEDSGVEMTTLRVDG

GAVKNNFLCQLQSDIIQTDIARPQVDETTALGSAYAAGLAVGYWDTVDELRDNWQVDEEF

SPEMDAGKADKMYARWDDAVDRSRDWAQEE

>tr|A0A8T8WFU1|A0A8T8WFU1_9EURY Glycerol kinase OS=Halobaculum magnesiiphilum OX=1017351 GN=glpK PE=3 SV=1

MPDTYVGAIDQGTTGTRFMVFDHAGQVVANAYEKHEQIYPEPGWVEHDPTEIWENTTTVV

TRGLADAGLEASQLEAIGITNQRETTVVWDADTGRPVHNALVWQDRRTTDRVEELQAADK

VEWIRGKTGLEADAYFSATKTEWILDNAEPLKLQSSRGGGLRERAEAGELLMGTIDSWLI

YNLTGNHITDVTNASRTMLYDIRELEWDDELLAEFDVPESMVPEVRPSSDEEYYGYTDPD

GFLGAEVPVAGALGDQQAAMFGQTCFDEGDAKNTYGTGSFYLMNTGTEAVESDHGLLTTI

GFQLSDEPVRYALEGSIFVTGAAIEWLEDVDLINNAAQTAELARSVDSTDGVYMVPAFTG

LGAPHWDGRARGTIVGMTRGTRKEHIVRATLEAIAYQTRDVAEAMEADSGVETTSLRVDG

GAVKNDFLCQLQSDIIQTEIARPEVDETTALGSAYAAGLAVGYWDSVDDLRQNWQVDREF

TPEMDAADADAMYDRWGDAVERSRNWATED

>tr|A0A8T8LJU0|A0A8T8LJU0_HALSD Glycerol kinase OS=Halorubrum sodomense OX=35743 GN=glpK PE=3 SV=1

MTQYVGAIDQGTTGTRFMVFDHEGQVVANAYEQHEQIYPNPGWVEHDPMEIWENTQQVVL

DGLADAGLEADQLDAIGITNQRETTIVWDKDSGRPVHNALVWQDRRTTDRVEELQEADKV

EEIREKTGLEADAYFSATKTEWILDNAEPLKMQSSRGGDLRDRARAGELVMGTIDSWLIY

NLTGNHITDVTNASRTMLYNIRELEWDDELLDEFDVPKEMVPEVRPSSDEDYYGHTDPDG

FLGEEVPVAGALGDQQAALFGQTCFDEGDAKNTYGTGSFYLMNTGTDAVESDHGLLTTIG

FQMSGEPVQYALEGSIFITGAAIEWLEDVDLINNAAQTAELARSVDSTDGVYMVPAFTGL

GAPHWDGRARGTIVGMTRGTSKEHIVRATLESIAYQTRDIAEAMEADSGVETTSLRVDGG

AVKNNFLCQLQSDIIQTEIARPEVDETTALGSAYAAGLAVGYWDNVDELRDNWQIDREFT

PEKSQGEVDKLYSRWDDAVERSKNWAIDEEEE

>tr|D8J4Q0|D8J4Q0_HALJB Glycerol kinase OS=Halalkalicoccus jeotgali (strain DSM 18796 / CECT 7217 / JCM 14584 / KCTC 4019 / B3) OX=795797 GN=glpK PE=3 SV=1

MTTEHFVGAIDQGTTGTRFMMFDHGGQVIANAYETHEQIYPEPGWVEHDPNEIWRNTQSV

MTDALSNAGLEAEQLAAIGVTNQRETTLLWDADTGQPIHNALVWQDRRTTDRVEELQEAG

MAETIREKTGLEVDAYFSATKAEWILDNADPIKTQRARPADLRERAADGEIKFGTIDTWL

IEKLTGNHITDVTNASRTMLYDIHEGDWDEELLDEFGIAREMLPEVRPSSDENHYGTTDP

DDFLGAEVPVAGALGDQQAALFGQTCFDPGDAKNTYGTGSFFLMNTGEEAVESEHGLLTT

VGFQRSGEPIQYALEGSIFITGAAIEWLVDVELIEDPAETESLARSVDSTDGVYLVPAFT

GLGAPHWDGRARGTIVGMTRGTTKEHIVRATLESIAYQTRDVAEAMEADSGIEMSSLRVD

GGAVKNNFLCQLQADSIGSEIVRPVVDETTALGSAYAAGLAVGYWETADELRDNWQVDRE

FAPEMASTEADRKYGRWQEAVERSLNWAQEE

>tr|A0A8U0HYX6|A0A8U0HYX6_9EURY Glycerol kinase OS=Halorussus limi OX=2938695 GN=glpK PE=3 SV=1

MVFDHDGTVVTDAYETHEQIYPEPGWVEHDPLEIWENTKSVVRAALDDAGLSADQLAAIG

VTNQRETTLLWDRDTGKPVHNAIVWQDRRTTDRVEQLESEGKVEEIRAKTGLEADAYFSA

TKAEWLLDNADPIKTQRARPADMKERAAEGEILFGTIDSWLIYNLTGNHITDVTNASRTM

LFDIHEMEWDDDLLREFDVPRESLPEVRPSSDEDYYGTTDPDGFLGAEIPVAGALGDQQA

ALFGQTCFDAGDAKNTYGTGSFFLMNTGNEAVDSDHGLLTTVGFQRSGEPVQYALEGSIF

VTGAAIEWLEDMTLIEDAAETEALARSVDSTDGVYVVPAFTGLGAPHWDQRARGTIVGMT

RGTRREHVVRATLESIAYQTRDVAEAMVADSDIEMESLKVDGGAVKNNFLCQLQADIIGT

EIARPEVDETTALGSAYAAGLAVGYWDDPDELRQNWRVDREFAPEMDRDEADRMYDRWGD

AVERSTDWARDGGDD

>tr|A0A1H1GFV6|A0A1H1GFV6_NATTX Glycerol kinase OS=Natronobacterium texcoconense OX=1095778 GN=glpK PE=3 SV=1

MTARTYVGAIDQGTTGTRFIVFDHEGQVVANAYETHEQIYPEPGWVEHDPVEIWENTKTV

VTEALGQAGISPDQLAAIGVTNQRETTVLWDADSGKPVYNAIVWQDRRTTDRIEELEDEG

KADWIREKTGLEPDAYFSATKAEWLLEEGDPIKMERARPADVRDRAESGDVLFGTIDSWL

IYNLTGNHVTEVTNASRTMLYDIHDCEWDDELLEEFSIPREMLPEVRPSSDDATYGSTDP

DGFLEAEVPVAGALGDQQAALFGQTCFDAGDAKNTYGTGSFVLMNTGEEAVESDHGLLTT

IGFQRSGEPVQYALEGSIFVTGAAIEWLEDVDLIADPAETAELARSVDSTDGVYVVPAFT

GLGAPHWDQRARGTIVGMTRGTRKEHIVRATLESIAYQTRDVTEAMEADSGIEMTSLKVD

GGAVKNNYLCQLQSDIIGSEIVRPIVDETTALGSAYAAGLAVGYWDDVDSLRNNWQVDRE

FEPDQDDDVDAKYGRWQDAVERSRDWANDGGGE

>tr|A0A5D5AJD6|A0A5D5AJD6_9EURY Glycerol kinase OS=Natrialba swarupiae OX=2448032 GN=glpK PE=3 SV=1

MTEDTYVGAIDQGTTGTRFIVFDHDGEVVANAYEKHDQYYPEPGWVEHDPMEIWTNTKDV

ITQALGWADIGPEQLAAIGVTNQRETTLLWDADTGTPIYNAIVWQDRRTTERIETLEENG

AVETIREKTGLEPDAYFSATKAEWLLEEADPIKMERARPSDVRDRAEAGEILFGTIDSWL

IYNLTGEHVTEVTNASRTMLYNIHDLEWDDDLLAEFSIPETILPEVRPSSDEATYGTTDP

DGFLGAEIPVAGALGDQQAALFGQTCFDPGDAKNTYGTGSFFLMNTGDDAVESDHGLLTT

IAYQRSGEDVQYALEGSIFVTGAAIEWLEDVSLIDDPGETAELARSVDSTDGVYVVPAFT

GLGAPHWDQRARGTIVGMTRGTRREHIVRATLESIAYQTRDVAEAMEGDSGIEMTSLKVD

GGAVKNNFLCQLQSDVIGSQIVRPVVDETTALGAAYAAGLAVDYWADLEQLRNNWRVDRE

FEPEIDPDVADQRHERWIDAVERSRGWAREES

>tr|A0A9E7SUL9|A0A9E7SUL9_9EURY Glycerol kinase OS=Natronosalvus rutilus OX=2953753 GN=glpK PE=3 SV=1

MTDTYVGAVDQGTTGTRFMVFDHSGQVVANAYEKHEQIYPEPGWVEHDPMEIWENTKSVI

TSALGQAGISPDQLEAIGVTNQRETTLLWDAESGKPVHNALVWQDRRTTSRVEQLEEDGM

VDTIREKTGLEADAYFSATKAEWLLENADPIKMERTRPSDIQDRAAEGEVLFGTIDTWLI

YNLTGNHITEVTNASRTMLYNIHDLEWDDELLEEFDVSREMLPEVRPSSDEDTYGTTDPD

GFLEAEIPVAGALGDQQAALFGQTCFDAGEAKNTYGTGSFFLMNTGEEAVESEHGLLTTI

GFQRSGEPVQYALEGAIFITGAAIEWLEDMTLIDDPAETAELARSVDSTDGVYFVPAFTG

LGAPHWDQRARGTIVGMTRGTRKEHVVRATLESIAYQTRDVAEAMEADSGIEMQDLKVDG

GAVKNNYLCQLQSDIIGSEISRPVVDETTALGSAYAAGLAVGYWGDVEGLRDNWQVDREF

TPEMDPEKADRMYSRWSDAVERSLGWATDGGD

>tr|A0A1I6RVQ3|A0A1I6RVQ3_9EURY Glycerol kinase OS=Halostagnicola kamekurae OX=619731 GN=glpK PE=3 SV=1

MTDPTYVGAIDQGTTGTRFIVFDHGGQVVANAYEKHEQIYPEPGWVEHDPMEIWDATKSV

VTTALGQAGISPDQLEAIGVTNQRETTLLWDADSGRPVHNAIVWQDRRTTDRVEQLESEG

MVEEIREKTGLEADAYFSATKAEWLLENADPIKLERSRPEDIRDRAAQGEVLFGTIDSWL

IYNLTGNHITEVTNASRTMLYNIHDLEWDDDLLAEFGVPEEMLPEVRPSSDDQTYGTTDP

DGFLEAEVPVAGALGDQQAALFGQTCFDPGEAKNTYGTGSFFLMNTGEEAVESDHGLLTT

IGFQRSGEPVQYALEGSIFITGAAIEWLEDVSLIDDPAETAELARSVDSTDGVYVVPAFT

GLGAPHWDQRARGTILGMTRGTRKEHIVRATLESIAYQTRDVAEAMEADSGIEMTSLKVD

GGAVKNNYLCQLQADIIGSEISRPVVDETTALGSAYAAGLAVGYWDDVEGLRDNWQVDRE

FEPEMNADAADRMYARWSDAVDRSRDWARDGEE

>tr|A0A1S8ATY5|A0A1S8ATY5_9EURY Glycerol kinase OS=Natrinema saccharevitans OX=301967 GN=glpK PE=3 SV=1

MPDTTYVGAVDQGTTGTRFIVFDHEGQVVANAYETHEQIYPEPGWVEHDPMEIWENTTAV

IQQALGRAGIAPSQLAAIGVTNQRETTVLWDADSGKPVHNAIVWQDRRTTDRVESLEAEG

KVAAIREKTGLEADAYFSATKAEWLLDNADPIKLERARPEDIRDRAEKGDVLFGTIDTWL

IYNLTGNHVTEVTNASRTMLYDIHDLEWDEDLLAEFSIPEAMLPEVRPSSDDDTYGTTDP

DGFLEAEVPVAGALGDQQAALFGQTCFDAGDAKNTYGTGSFFLMNTGDEAVESDHGLLTT

IGFQRSGEPVQYALEGSIFVTGAAIEWLEDMTLIEDPAETAELARSVDSTDGVYVVPAFT

GLGAPHWDQRARGTIVGMTRGTRKGHVVRATLESIAYQTRDVAEAMEADSGIEMTSLKVD

GGAVKNNFLCQLQSDIIGSEIVRPVVDETTALGSAYAAGLAVGYWDDPDELRSNWQVDAE

FEPEMDPDRADRRYERWNDAVERSRDWARDEEE

>tr|A0A8J8GNX4|A0A8J8GNX4_9EURY Glycerol kinase OS=Haloterrigena gelatinilytica OX=2741724 GN=glpK PE=3 SV=1

MTESTYVGAVDQGTTGTRFIVFDHGGQVVANAYETHEQIYPEPGWVEHDPMEIWENTKSV

ITQALGQAGISPDQLEAIGVTNQRETTLLWDADSGRPVHNAIVWQDRRTTDRVEQLEAEG

QVETIREKTGLEADAYFSATKAEWLLDNADPIKLERSRPEDIRDRAEKGDVLFGTIDSWL

IYNLTGEHITEVTNASRTMLYNIHDLEWDDDLLAEFDVPKEMLPEVRPSSDDDTYGTTDP

EGFLEAEIPVAGALGDQQAALFGQTCFDAGDAKNTYGTGSFFLMNTGSEAVTSDHGLLTT

IGFQRSGEDVQYALEGSIFITGAAIEWLEDLSLIDNPAQTAELARSVDSTDGVYVVPAFT

GLGAPHWDQRARGTIVGMTRGTRKEHVVRATLESIAYQTRDVAEAMEADSGIEMTSLKVD

GGAVKNNYLCQLQSDIIGSEIVRPVVDETTALGSAYAAGLAVGYWDDVDSLRDNWQIDRE

FEPEMDADRADNRYARWTEAVDRARDWARDDEE

>sp|D4GYI5|GLPK_HALVD Glycerol kinase OS=Haloferax volcanii (strain ATCC 29605 / DSM 3757 / JCM 8879 / NBRC 14742 / NCIMB 2012 / VKM B-1768 / DS2) OX=309800 GN=glpK PE=2 SV=1

MSGETYVGAIDQGTTGTRFMVFDHDGKVVANAYEKHEQIYPEPGWVEHDANEIWDNTKQV

IDAALSSAGLDAEQLEAIGITNQRETTLVWDRETGQPIHNAIVWQDRRTTDRIETLEAEG

KTDDVRAKTGLEPDAYFSATKAEWLLDNSDPIKLQRSRPEDIRDRAADGELAFGTIDTWL

IYNLTGNHITDVTNASRTMLFNIHDMEWDDELLDEFNVPRELLPEVRPSSDDDYYGTTDA

DGFLGAEVPVAGALGDQQAALFGQTCFDAGDAKNTYGTGSFMLMNTGDEAVMSEHGLLTT

VGFQRSGEPVQYALEGSIFITGAAIEWLEDMTLIDNAAESEKLARSVESTDGVYFVPAFT

GLGAPHWDQRARGTIVGMTRGTRREHIVRATLESIAFQTRDVAEAMESDSEIDLSSLRVD

GGAVKNNFLCQLQSNILDTEIVRPQVDETTALGAAYAAGLAVGYWETLDELRENWQVDRE

FAPKDPQNVEHRYGRWKEAVDRSLDWAREE

>tr|A0AAV3T1V8|A0AAV3T1V8_9EURY Glycerol kinase OS=Salarchaeum japonicum OX=555573 GN=glpK PE=3 SV=1

MYVGAIDQGTTGTRFLVFDRDASVVAGAYETHEQHYPEPAWVEHDPGELWANTQSVVESA

LADAGIAPADLAAIGVTNQRETTVLWDAETGDPVHDAIVWQDRRTTERIEGLSDDLSAYV

QETTGLEPDAYFSASKLEWLLENADPIEPTRSATRSVRERAESGEVLFGTIDSWLIYKLT

GEHVTDVTNASRTMLYDIHDCAWDDRLLAEFDVPRACLPDVRPSSDDEYYGYTDPGGFLG

AQVPVAGALGDQQAALFGQACFDPGDAKNTYGTGSFFLLNTGETPVESTHGLLTTIGFQR

RGEPVQYALEGSIFATGAAIEWLNDVSLVEDAGETESLARAVDGTDGVYVVPAFTGLGAP

HWDPRARGTIVGLTRGTRREHVVRATLEAIAYQTRDVAEAMLADSDVSLSTLRVDGGAVK

NDFLCQLIADVLDTDIDRPVVDETTALGAAYAAGLAADYWRDLDALRENRRTDRAFTPRD

GDWDAKYDRWQDAVERSRDWATESP

>tr|A0AAE3FQW6|A0AAE3FQW6_9EURY Glycerol kinase OS=Natranaeroarchaeum aerophilus OX=2917711 GN=glpK PE=3 SV=1

MTQDTYVGAVDQGTTGTRFMVFDHSGQVVANAYEKHEQIYPEPGWVEHDPREIWENTKQV

IQDALNDAGLTPDQLEAIGVTNQRETTLLWDADTGRPLHNALVWQDRRTTDRIETLQEEG

KADDVQAKTGLEPDAYFSATKAEWLLDNADPIKTQRARPADVRERAAEGDVLFGTIDTWV

IYNLTGNHITDVSNASRTMLFDIHEMEWDDELCEEFRVPKEMLPEVRPSSDEKTYGSTDS

DGFLNAEVPVAGALGDQQAALFGQTCFEAGDAKNTYGTGSFFLMNTGNEAVTSENGLLTT

VGFQRSGEEVQYALEGSIFITGAAIEWLNDMDLIEDAAETEELARSVDSTDGVYFVPAFT

GLGAPHWDQRARGTIVGMTRGTRREHIVRATLESIAYQTRDVAEAMEADSGIDMQTLRVD

GGAVKNNYLCQLQSDIIDTDIARPEVDETTALGSAYAAGLAVGYWSDLDELTANWQIDRE

FEPEMSNEEADKLYSRWSDAIDRSRDWARDGGD

>tr|A0AAE3K8L0|A0AAE3K8L0_9EURY Glycerol kinase OS=Natronocalculus amylovorans OX=2917812 GN=glpK PE=3 SV=1

MADNTYVGAIDQGTTGTRFMVFDHSGKVIANAYEKHEQIYPEPGWVEHDAMEIWENTKSV

INTALDDALIDAEQLEAIGVTNQRETTVMWDKDTGSPIGNAIVWQDRRTTDRIETLEADG

KKDDVQQKTGLEPDAYFSATKAEWLLDNTDPIKLQRSRPEDVRDRAEEGEILFGTIDTWV

IYNLTGNHITDVTNASRTMLFNIHDMEWDDELLEEFNVPRATLPEVRPSSDEDTYGSTDA

DGFLGAEVPVAGALGDQQAALFGQTCFDAGDAKNTYGTGSFMLMNTGDEAVMSEHGLLTT

VGFQRSGQPVQYALEGSIFITGAAIEWLEDMTLISDAMESEKLARSVDSTDGVYFVPAFA

GLGAPHWDQRARGTIVGMTRGTRREHIVRATLESIAFQTRDVAEAMEEDSGIDLQSLRVD

GGAVKNNFLCQLQSNIINTDIARPVVDETTALGSAYAAGLAVGYWDTIDELRDNWQIDRE

FSPKDTSDVDARYDRWQEAVKRSLDWARGGD

>tr|A0A830EYD1|A0A830EYD1_9EURY Glycerol kinase OS=Halarchaeum grantii OX=1193105 GN=glpK PE=3 SV=1

MADTYVGSIDQGTTGTRFMVFDHSGEVVANAYEKHEQIYPEPGWVEHNPMEIWENTQEVV

TRGLEEAGLDAEQLEGIGITNQRETTLVWDRESGKPVHNALVWQDRRTTDRVEELEAADK

VEWIREKTGLEADAYFSATKTEWILDNAEPLKLQSSRSQDLRDRARDGELLMGTIDAWLI

YNLTGEHITDVTNASRTMLFDIHEMEWDEELLAEFDVPAEMLPEVRPSSDENYYGHTDSD

GFLGAEVPVAGALGDQQAALFGQTCFDAGDAKNTYGTGSFYLMNTGNEAVESEHGLLTTV

GFQMSGEPVQYALEGSIFITGAAIEWLEDVDLIDNAAQTAELARSVDSTDGVYMVPAFTG

LGAPHWDGRARGTIVGMTRGTKKEHIVRATLESIAYQTRDIAEAMEADSGVETTSLRVDG

GAVKNNFLCQLQADIIQTEIARPEVDETTALGSAYAAGLAVGYWDTIDELRSNWQVDREF

DAEMSQSDADRMYGRWDDAVERSLDWAQEE

>tr|A0A554MVE3|A0A554MVE3_9EURY Glycerol kinase OS=Haloglomus irregulare OX=2234134 GN=glpK PE=3 SV=1

MPENTYVGAIDQGTTGTRFMIFDHAGQVVANAYEKHEQIYPEPGWVEHDPMEIWENTKTV

VSRALREGGLDPGQLAGLGITNQRETTVIWDAETGTPIYNALVWQDRRTTARVEELQAAD

KVEWIREKTGLECDAYFSATKAEWLLDNADPIKLERVRPQDIRDRAEDGQLRMGTIDSWL

IYKLTGRHVTDVTNASRTMLYNIREMEWDDELLDEFNVPVQLLPEVRPSSDDDYYGHTDP

EGFLGAEVPVAGALGDQQAALFGQTCFDEGDAKNTYGTGAFFLMNTGEEPVRSDHGLLTT

VGFQRSGEPVQYALEGSIFVAGAAIEWLEDVDLVDDPTETAELARSVESTDGVYVVPAFT

GLGAPHWDGRARGTIVGMTRGTRREHLVRATLESIAYQTRDLAEAMEADSDIEMSTLRVD

GGAVKNNFLCQLQSDIIGTDIARPVVDETTALGSAYAAGLAVGYWDDTDELRENWQVDRQ

FSPRMDRPRANEMYARWDDAVERSLDWAQDEGGE

>tr|A0AAJ4R8Y3|A0AAJ4R8Y3_9EURY Glycerol kinase OS=Halosegnis longus OX=2216012 GN=glpK PE=3 SV=1

MHYVAAIDQGTTGTRCLLVSHDGAVVGQAYETHEQQYPQPGWVEHDPTVLWENTKTVVTD

ALADAGLAASDLAALGVTNQRETTVLWDAATGEPIHNAIVWQDRRTTERIEQLETEWIRD

RTGLEPDAYFSASKLEWLLDEVEGARERAEAGELRFGTVDSWLVSKLTGEHVTDVTNASR

TMLFDIHDLRWDGDLLAEFDVPRAVLPTVVPSSDPESYGTTSADGFLGAEIPVAGVLGDQ

QAALFGQTCFEAGSAKNTYGTGSFLLVNTGPDPVESDHGLLTTVAFQRAGEEPRYALEGS

IFTTGAAIEWLEDVSLVESPAETETLARSVDSIDGVYVVPAFSGLGAPHWDGRARGTILG

LTRGTGREHIARATLESIGFQTHDVLEAMAADTGETIDRLRVDGGAVQNDLLCELQATIA

DCTVARPAVDETTALGAAYAAGLAVGYWEDLDALQEQHRTERVFRPSETGDMDGRYERWN

EAVDRARDWAREER

>tr|M1XTK0|M1XTK0_NATM8 Glycerol kinase OS=Natronomonas moolapensis (strain DSM 18674 / CECT 7526 / JCM 14361 / 8.8.11) OX=268739 GN=glpK1 PE=3 SV=1

MATSYVGAIDQGTTGTRFMVFDHSGHVIANAYEQHEQIYPEPGWVEHDPVEIWENTKSVV

LSGLDEAGLDAEQLAALGITNQRETTIVWDRESGKPVHNALVWQDRRTTDRVEELQEAGK

VEEIREKTGLECDAYFSATKTEWILDNAEPLKLQSSRSRDLRSRARDGELLMGTIDSWLI

YNLTGNHITDVSNASRTMLYNIEGMHWDEELLAEFDVPEAMLPEVRPSSDESLYGHTDAD

GFLGAEVPVAGALGDQQAALFGQTCFDAGDAKNTYGTGSFYLMNTGNEAVSSDHGLLTTV

GFQLSGEPVQYALEGSIFVTGAAIEWLEDVDIINNAAQTAELASSVDSTDGVYMVPAFTG

LGAPHWDGRARGTIVGMTRGTGKEHIVRATLESIAYQTRDIAEAMEADSGVETTTLRVDG

GAVKNNFLCQLQADIIQTEIARPEVDETTALGSAYAAGLAVGYWDDLDELRSNWQVDREF

DAEMESEQADGMYGRWDDAVERSLDWAQEE

>tr|A0A8J8PB72|A0A8J8PB72_9EURY Glycerol kinase OS=Halonotius terrestris OX=2487750 GN=glpK PE=3 SV=1

MSETYVGSIDQGTTGTRFMVFDHDGGVVSQAYEKHEQIYPEPGWVEHDPMEIWENTKLVI

NEALDKEGIDPEQLEALGVTNQRETTIVWDKETGKPVHNALVWQDRRTTDRVEEIQDEST

DTLTVETIREKTGLECDAYFSATKTEWILDNADPIKLQRSRPQDVRDRAEEGELLMGTID

TWVIYNLTGNHITDVSNASRTMLYNIEDLEWDDELLEEFRVPESMLPEVRPSSDDDTYGS

TDPDGFLGAEVPVAGALGDQQAALFGQTCFDAGDAKNTYGTGSFMLMNTGNEAVPSEHGL

LTTIGFQRTGGDVQYALEGAIFITGAAIEWLEDMTLIEDAAESEELARSIDSTDGVYVVP

AFTGLGAPHWDQRARGTIVGMTRGTRREHIVRATLESIAYQTKDVAEAMVEDADIDLSSL

RVDGGAVKNNFLCQLQSNIIGTEIVRPVVDETTALGSAYAAGLAVGYWDTLDELRNNWQV

DRKFPVDEDADIEVNYDRWKDAVERSEGWAQDEGGD

>tr|A0A6B0T515|A0A6B0T515_9EURY Glycerol kinase OS=Halovenus carboxidivorans OX=2692199 GN=glpK PE=3 SV=1

MSDTYVGAIDQGTTGTRFMVFDHGGQVVANAYTKHEQIYPEPGWVEHDAGEIWQNTKEVV

LEGLNEAGLDAEQLEALGITNQRETTVVWDAETGEPIHNAIVWQDRRTTDRVEELQEADK

VEWIREKTGLEADAYFSATKAEWLLENADPVGTDRGGDALEDRARNGELLMGTIDTWLIY

NLTGNHITDVSNASRTMLYNVRDLEWDDELLEEFEIPRAMLPEVRPSSDENTYGYTDAGG

FLGAEIPVAGALGDQQAALFGQTCFDAGDAKNTYGTGSFYLMNTGNEAVASDHGLLTTIG

FQLSGEPVQYALEGSIFITGAAIEWLEDLELISDAAETAGLARSVDSTDGVYFVPAFTGL

GAPHWDGRARGTIVGMTRGTRKEHLVRATLEAIAYQTRDVAEAMEADSGVETTSLRVDGG

AVKNNFLCQLQADILQTEIVRPEVDETTALGSAYAAGLAVGYWEDLDSLRQNWHVDEDFQ

AERNRQEVDEKYDRWKDAVERSLNWAEDEGE

>tr|A0A6B0GNB0|A0A6B0GNB0_9EURY Glycerol kinase OS=Halomarina oriensis OX=671145 GN=glpK PE=3 SV=1

MEDTYIGAIDQGTTGTRFMVFDHAGQVVANAYEKHEQIYPEPGWVEHDPIEIWENTQSVV

QAALEQGGIDATQLEAIGITNQRETTLVWDAETGRPVHNALVWQDRRTTDRVEALQDQDK

VEWIREKTGLEADAYFSATKTEWILDNAEPLKLSTSRTQDLRDRAEAGELLMGTIDSWLI

YNLTGNHITDVTNASRTMLYDIEALDWDAELLEEFDVPEAMLPEVRPSSDEEFYGTTDPD

GFLDAEVPIAGALGDQQAALFGQTCFDVGDAKNTYGTGSFYLMNTGNEAVSSDHGLLTTI

GFQMSGEPVQYALEGSIFVTGAAIEWLEDVDLINNAAQTAELARSVDSTDGVYMVPAFTG

LGAPHWDGRARGTIVGMTRGTRKAHIVRATLEAIAYQTRDIAEAMENDSGVETTSLRVDG

GAVKNNFLCQLQSDIIQTDIARPEVDETTALGSAYAAGLAVGYWDTIDELRDNWQVDREF

EPEMTADDADRMYARWDDAVERSLNWATEE

>tr|A0A2I8VIU7|A0A2I8VIU7_9EURY Glycerol kinase OS=Salinigranum rubrum OX=755307 GN=glpK PE=3 SV=1

MSQDTFVGAIDQGTTGTRFMVFDHAGEVVANAYEKHEQIYPEPGWVEHDANEIWRNTKHV

INTALDEAGLSSDQLEAIGITNQRETTVVWDAETGKPIHNALVWQDRRTTDRVEEIQAED

KVEWIREKTGLECDAYFSATKAEWILDNADPIKLSRSRPEDVRDRAESGELMMGTIDSWL

IYKLTGNHITDVTNASRTMLYNIHDNEWDDELLEEFNVPAAMLPEVRPSSDDEFYGTTSD

VGFLDADIPVAGALGDQQAALFGQTCFDEGDAKNTYGTGSFMLMNTGEEAVASEHGLLTT

IGFQRSGEPVQYALEGAIFITGAAIEWLEDMTLIENAAETEELARSVDSTDDVYVVPAFT

GLGAPHWDGRARGTIVGMTRGTRREHVVRATLESIAYQTRDVAEAMEADSGIDMGALRVD

GGAVKNNFLCQLQADIIDTDIVRPVVDETTALGSAYAAGLAVGYWETLDELRDNWQVDRE

FEVDPDADYESNYGRWKEAVARAEDWARDGGD

>tr|A0A1H0XR87|A0A1H0XR87_9EURY Glycerol kinase OS=Halopelagius longus OX=1236180 GN=glpK PE=3 SV=1

MSEERYIGAIDQGTTGTRFMVFDHDGQVVANAYQKHEQVYPEPGWVEHKPQEIWENTKEV

IDAALTEAGIGSEQLDAIGITNQRETTLIWDRESGKPIHNAIVWQDRRTTDRVEQLQDEG

KEGWIREKTGLEPDAYFSATKAEWLLDNTDQIKLQRMRPDDVRDRAEQGELLFGTIDSWL

IYKLTGEHITDVTNASRTMLFNIHEMDWDEELLEEFGVPEAILPEVRPSSDENHYGYTDA

DGFLGAEVPVAGALGDQQAALFGQTCFDEGDAKNTYGTGSFMLMNTGDEAVESEHGLLTT

VGFQRSGEPVKYALEGSIFITGAAIEWLEDMTLIDDAAETEELARSVDSTDGVYFVPAFA

GLGAPHWDQRARGTIVGMTRGTRREHVVRAVLESIAFQTRDVAEAMESDSGIDLTTLRVD

GGAVKNNFLCQLQANIVGTDIARPVVDETTALGAAYAAGLAVGYWETVDELRDNWHVDSE

FQPESPGDVEQRYGRWTDAVERSLDWARDGGE

>tr|A0A1I2VDC5|A0A1I2VDC5_9EURY L-xylulokinase OS=Halopelagius inordinatus OX=553467 GN=SAMN04488063_3144 PE=3 SV=1

MTDERLLLGVDAGLTNVKAAVFDAEGRERAVAVRPTPNERPEPNRVERDLSAFFRVTCET

IREAVGSPDVDADRIAGVGIAGHGHGLYALDEAGDPIRDGITSLDSRASDVVASWREDGR

LEEVRDEIGYEPFVADPLSLLGWMKREEPDAYDDIDRILSCKDYLKYRLTDVVCTDEMEA

SVFYDVSRETYSETAFEILGLEDRFDALPDVVPSWETCGEVTAEASEKTGLPVGTPVASG

LHDVGAVALGAGAFRTGQATLIVGTWGQSIYVTDDPDPGSEGLSRRFLRDSWLRYRGTRS

AAAAVDWFTDEYCGDWRDAADSEAAFYDRLNDSVADVPIGSNGLLFLPYLRGSTDDPDAR

GGFVGLTAEHGRPEMLRSIYEGVALSSIDRLRELTAGKRLTDVRLGGGGAKSRVWSQMFA

DVLGEQVVVPSGEETGARGVAICAGIAAGLYEDHETAVERTVGVARRHDPTEARTEAYRS

VRDAFRTTVSGTEATWEKLKAIEQENQTDDDR

>tr|A0A1G9W1X0|A0A1G9W1X0_9EURY Glycerol kinase OS=Halogranum gelatinilyticum OX=660521 GN=glpK PE=3 SV=1

MVDTYVGSIDQGTTGTRFMVFDHSGQVVANAYKKHEQIYPNPGWVEHDPTEIWENTKEVV

VEGLENAGLDASQLEALGITNQRETTIVWDKESGRPVHNALVWQDRRTTDRVEELQDEGK

VEDIREKTGLEADAYFSATKTEWILDNAEPLKLQSSREGNLRKRAEEGELLMGTIDAWLI

YNLTGNHITDVTNASRTMLYNIRDLEWDDELLDEFGVPKEMVPEVRPSSDENHYGHTDPD

GFLGEEIPVAGALGDQQAALFGQTCFDKGDAKNTYGTGSFYLMNTGNEAVASDHGLLTTI

GFQMSGEPVQYALEGSIFITGAAIEWLEDVDLINNAAQTAELARSVDSTDGVYMVPAFTG

LGAPHWDGRARGTIVGMTRGTRKEHIVRATLEAIAYQTRDVAEAMEADSGVELTSLRVDG

GAVKNNFLCQLQSDIIQTDIARPEVDETTALGSAYAAGLAVGYWDTVDELRDNWQIDREF

TPEKSADDVDKLYDRWHDAVDRSLDWAQEE

>tr|A0A8J7ZHN0|A0A8J7ZHN0_9EURY Glycerol kinase OS=Halorubellus sp. JP-L1 OX=2715753 GN=glpK PE=3 SV=1

MTDTTYVGAIDQGTTGTRFIVFDHSGQIAANAYTKHEQHYPEPGWVEHDPMEIWENTKVV

VRDALDDAGIGADQLAGLGITNQRETTLLWDADTGKPVHNALVWQDRRTTDRVEALQDAG

KVEAIREKTGLEADAYFSATKAEWLLDNADPIKMERTRPDDVRQRASRGDVLFGTIDTWL

VWKLTGNHVTDVTNASRTMLYDIRKTEWDDDLLAEFDVPRETLPEVRPSSDDDYYGYTDA

DGFLDAEVPVAAALGDQQAALFGQTCFDEGDAKNTYGTGSFFLMNTGEEAVRSDHGLLTT

IGFQRSGEPVQYALEGAIFATGAAIEWLEDVGFVDDPAETAELARRVDSTDGVYVVPAFT

GLGAPHWDQRARGTIVGMTRGTRKEHVVKATLESIAYQTRDVAEAMEADSGIEMTTLRVD

GGAVKNNYLCQLQSDIIGSEIVRPEVDETTALGAAYAAGLAVGYWDTVDELRENWQVDRE

FQPAMQPDEADAKYARWDDAVDRSLDWAVDDGGS

>tr|A0A0W1REE8|A0A0W1REE8_9EURY Carbohydrate kinase OS=Haloprofundus marisrubri OX=1514971 GN=AUR64_04180 PE=3 SV=1

MFIGIDIGHTNLKAVAYDSEWEVVGSHGIEAGMKHPSDDRHEIPIEERWDLTMDCLASLT

DQISQSGDIDCIGLTGGGGGLYPLDENEEPFMNGIPLLDERAKGVLRRWKEDGTFNDISE

RTGIPIPPGAALLSLRWLKENEPENYDRIAHIFNLKDIVRYKLTGEKALEISDATFSFTN

HETQDYDDALFDLAGVEEKRDALPELAGSSYEIAGYTTEEVQRDTDIPEGTPVITGAHDA

CANALGVGAIEENVVTTAGGTWSLSTMVLDSPTVDLDSWCCENFLERGTWMLEIAQPTGT

VSLDWFVDEYFEEEKQQAEEENQRVWSIIEEQLEDVSTTALFHPFLLGNPYGYLYQDNAT

GSFTGLTNQDGRFEMLRAIYEAISFMHRWQIEQFERELRVNEVRFTGGAAKSKFWAQMFA

DVFDTKITMTEKKESGCFGAAMLAAIGIGEISGLEETTEYVTVTEEYSPRGDEQREHFEQ

KYNAFTEMAVLLEEIWNIHEGLRSSELATYN

>tr|A0A4D7HB98|A0A4D7HB98_9EURY Glycerol kinase OS=Haloprofundus sp. MHR1 OX=2572921 GN=glpK PE=3 SV=1

MATETYVGAIDQGTTGTRFMVFDHSGRVVTSAYEKHEQIYPEPGWVEHDPAEIWEKTQSV

ITSALQKADLTADQLEAIGITNQRETTLLWDADSGTPVHNAIVWQDRRTTDRVEELEAED

KVEWIRGKTGLEADAYFSATKAEWLLDNADPMKMQRARPADVRERAEDGEILFGTIDTWL

IYKLTGNHITDVTNASRTMLFDIHEMEWDEELCEEFDVPMAMLPEVRPSSDDNYYGTTDS

EGFLGSEVPVAAAFGDQQAALFGQTCFDEGDAKNTYGTGSFMLMNTGEEAVESEHGLLTT

VGFQRSGEPVQYALEGAIFVTGAAIEWLEDMEIISNASESETLARSVDSTDGVYFVPAFT

GLGAPHWDGRARGTIVGMTRGTRREHVVRATLESIAFQTKDVADAMEADSGIEMTTLRVD

GGAVKNNFLCQLQANIVDSTIVRPQVDETTALGSAYAAGLAVGYWETLDELRDNWQVDRE

FEAEDGKNVERRHERWQEAVKRSLDWARDGSD

>tr|A0A830FP33|A0A830FP33_9EURY Glycerol kinase OS=Halocalculus aciditolerans OX=1383812 GN=glpK PE=3 SV=1

MSAYVGAIDQGTTGTRFMVFDHDGRVVASAYETHEQSYPEPGWVEHDPVEIWENTQRVMV

DALASADLDAAALDAIGVTNQRETTVLWDAHTGEPVYDAVVWQDRRTTERVEELDADGWH

DYIRATTGLEPDSYFSGTKVEWLLDNADPIKLQRTRPEDVRDRAEDGDVLFGTMDSWLVY

NLTGAHVTDVTNASRTMLYDIHAGEWDDDLLAELRVPGAMLPEVRPSSDADYYGHTDPDG

FLGARVPVAGALGDQQAALFGQTCFAAGDAKNTYGTGSFMLLNTGAEAVESDHGLLTTVA

FQRSGDPVQYALEGSVFVTGAAIEWLADVGLIEDARETATLARSVDSTDGVYVVPAFTGL

GAPHWDQRARGTIVGMTRGTRREHVVRATLEAIAYQTRDVVEAMEADAGVDIDELRVDGG

AVKNNFLCRLQAGVLGSRIVRPEVDETTALGAAYAAGLATGYWESVEELRANWRVDRAFD

PDEDGVDGDWDARYDRWGDAVERAKHWAVEES
